# Neuroinflammation is associated with Alzheimer’s disease co-pathology in dementia with Lewy bodies

**DOI:** 10.1186/s40478-024-01786-z

**Published:** 2024-05-07

**Authors:** Janna van Wetering, Hanne Geut, John J. Bol, Yvon Galis, Evelien Timmermans, Jos W.R. Twisk, Dagmar H. Hepp, Martino L. Morella, Lasse Pihlstrom, Afina W. Lemstra, Annemieke J.M. Rozemuller, Laura E. Jonkman, Wilma D.J. van de Berg

**Affiliations:** 1 Department of Anatomy and Neurosciences, Section Clinical Neuroanatomy and Biobanking and Life Sciences O|2 building 13e55, Amsterdam UMC location Vrije Universiteit Amsterdam, De Boelelaan 1118, Amsterdam, 1081 HV The Netherlands; 2 Neurodegeneration, Amsterdam Neuroscience, Amsterdam, The Netherlands; 3 Department of Epidemiology and Biostatistics, Amsterdam UMC location Vrije Universiteit Amsterdam, De Boelelaan 1117, Amsterdam, The Netherlands; 4 Department of Neurology, Leiden University Medical Center, Albinusdreef 2, Leiden, 2333 ZA The Netherlands; 5 Department of Neurology, Oslo University Hospital, Oslo, Norway; 6 Department of Neurology, Amsterdam UMC location Vrije Universiteit Amsterdam, Amsterdam, De Boelelaan 1117 The Netherlands; 7 Alzheimer Center, Department of Neurology, Amsterdam UMC, Vrije Universiteit Amsterdam, Amsterdam Neuroscience, Amsterdam, The Netherlands; 8 Department of Pathology, Amsterdam UMC location Vrije Universiteit Amsterdam, De Boelelaan 1117, Amsterdam, The Netherlands

**Keywords:** Lewy body disease, Dementia with Lewy bodies, Alzheimer’s disease, Co-pathology, Neuroinflammation, Alpha-synuclein, Amyloid-beta, Phosphorylated-tau, Microglia, Astroglia, Post-mortem, Immunohistochemistry

## Abstract

**Background:**

Neuroinflammation and Alzheimer’s disease (AD) co-pathology may contribute to disease progression and severity in dementia with Lewy bodies (DLB). This study aims to clarify whether a different pattern of neuroinflammation, such as alteration in microglial and astroglial morphology and distribution, is present in DLB cases with and without AD co-pathology.

**Methods:**

The morphology and load (% area of immunopositivity) of total (Iba1) and reactive microglia (CD68 and HLA-DR), reactive astrocytes (GFAP) and proteinopathies of alpha-synuclein (KM51/pser129), amyloid-beta (6 F/3D) and p-tau (AT8) were assessed in a cohort of mixed DLB + AD (*n* = 35), pure DLB (*n* = 15), pure AD (*n* = 16) and control (*n* = 11) donors in limbic and neocortical brain regions using immunostaining, quantitative image analysis and confocal microscopy. Regional and group differences were estimated using a linear mixed model analysis.

**Results:**

Morphologically, reactive and amoeboid microglia were common in mixed DLB + AD, while homeostatic microglia with a small soma and thin processes were observed in pure DLB cases. A higher density of swollen astrocytes was observed in pure AD cases, but not in mixed DLB + AD or pure DLB cases. Mixed DLB + AD had higher CD68-loads in the amygdala and parahippocampal gyrus than pure DLB cases, but did not differ in astrocytic loads. Pure AD showed higher Iba1-loads in the CA1 and CA2, higher CD68-loads in the CA2 and subiculum, and a higher astrocytic load in the CA1-4 and subiculum than mixed DLB + AD cases. In mixed DLB + AD cases, microglial load associated strongly with amyloid-beta (Iba1, CD68 and HLA-DR), and p-tau (CD68 and HLA-DR), and minimally with alpha-synuclein load (CD68). In addition, the highest microglial activity was found in the amygdala and CA2, and astroglial load in the CA4. Confocal microscopy demonstrated co-localization of large amoeboid microglia with neuritic and classic-cored plaques of amyloid-beta and p-tau in mixed DLB + AD cases.

**Conclusions:**

In conclusion, microglial activation in DLB was largely associated with AD co-pathology, while astrocytic response in DLB was not. In addition, microglial activity was high in limbic regions, with prevalent AD pathology. Our study provides novel insights into the molecular neuropathology of DLB, highlighting the importance of microglial activation in mixed DLB + AD.

**Supplementary Information:**

The online version contains supplementary material available at 10.1186/s40478-024-01786-z.

## Background

The incidence of dementia is increasing rapidly as the population ages [1, 2]. Alzheimer disease (AD) and dementia with Lewy bodies (DLB) are the two most common subtypes of neurodegenerative dementia [3]. While AD and DLB have overlapping clinical features, AD is characterized by impairment in memory and learning, executive function and aphasia, whilst the core clinical features of DLB include visual hallucinations, cognitive fluctuations, rapid eye movement (REM) sleep behavior disorder (RBD) and parkinsonism [3, 4]. The clinical presentation of DLB can vary widely between patients in terms of disease onset, disease progression, treatment response and adverse side effects.

Protein misfolding, accumulation and aggregation are the primary pathological hallmarks of both DLB and AD [4]. In DLB, abnormal alpha-synuclein (α-syn) accumulates and aggregates in neurons, leading to the formation of Lewy bodies (LBs) and Lewy neurites (LNs), which are typically found in the brainstem, limbic and neocortical areas, spinal cord and peripheral nervous system [5]. AD is characterized by the deposition of extracellular amyloid beta (Aβ) plaques and intracellular phosphorylated tau (p-tau) tangles [6, 7]. Approximately 50–80% of DLB cases exhibit AD co-pathology, while circa 50% of AD cases show α-syn pathology in the amygdala and other limbic regions, as observed in clinicopathological studies [8–10]. On top of that, within Lewy body disorders (LBD), the amygdala is suggested to be prone to even initiate the development of α-syn pathology [11] and function as an incubator for coexisting pathologies such as Aβ and p-tau [12]. Accumulating evidence from disease models reports interactions between α-syn, p-tau and Aβ pathology, which together form a destructive feed-forward loop towards more severe and faster neurodegeneration [13–16]. Compared to separate pathology, the combination of α-syn, p-tau, and Aβ semi-quantitative pathology scores best predict cognitive decline, as measured by the MMSE [17]. In addition, DLB patients with AD co-pathology show a highly aggressive disease course with rapid cognitive decline compared to pure DLB cases [4, 18–20]. More importantly, aggregation of α-syn, p-tau, and Aβ pathology is associated with the activation of innate and adaptive immune responses in the elderly [21].

It is well established that, in AD, the innate immune system is activated in early stages of the disease, and reactive microglia and astrocytes surrounding Aβ plaques are frequently described [22–25]. Regional differences in glial activation have been reported in PD and DLB cases compared to controls, with some studies suggesting a higher activated microglial load, as measured by HLA-DR or CD68, in the amygdala [26], hippocampus [27, 28], transentorhinal cortex [27, 29], and temporal cortex [27, 30], whereas other studies did not describe an increase in the hippocampus [31] or neocortex [32]. Postmortem studies using Iba1 have reported the absence of microglial activity in DLB cases compared to AD cases or controls in the hippocampus [31] and cerebral cortex [30, 32]. Only two post-mortem studies of astroglial activation have reported an increase in GFAP expression in the temporal cortex [33] and pulvinar [34] in DLB cases compared to controls. The inflammatory response in DLB cases with AD co-pathology has not yet been elucidated, although clarifying these pathomechanisms serves several purposes, such as developing biomarkers to improve early diagnosis, predict disease progression, or to discover targets for early disease-modifying drugs [35]. Targeting glial cells has been proposed to be beneficial in early-stage neurodegenerative diseases, as neurodegeneration and neuronal cell death are irreversible and are thought to result from chronic neuroinflammation [36–38].

Recent cerebrospinal fluid (CSF) biomarker studies suggest that inflammation in DLB is related to AD co-pathology and is therefore less pronounced in pure DLB cases [39, 40]. For example, stratification of a DLB group with coexisting AD pathology in the CSF demonstrated higher levels of the glial marker YKL-40 [39], suggesting that the increase is related to AD neurodegeneration. In addition, a post-mortem study described that the number of activated microglial cells in PD was not associated with the number of LBs [41]. Moreover, one postmortem study in DLB reported that biochemical measures of CD200 and ICAM-1 correlated with AD plaque density, and found direct co-localization of microglia with AD plaques rather than with LBs [41, 42]. Interestingly, α-syn accumulation is also observed in activated astrocytes in PD and DLB which may suggest a direct link between α-syn accumulation and activation of the innate immune system [11, 43–45]. In addition, PET imaging studies with 11 C-PK11195, a marker of microglial activation [46], showed significantly higher binding in early DLB cases when compared to those with an advanced disease stage [47, 48]. Likewise, 11 C-PK11195 binding was increased in early AD, suggesting that microglial activation is an early event in both DLB and AD [49, 50]. However, not all studies distinguish between pure DLB and mixed DLB + AD cases, i.e. cases with mixed DLB and AD pathology, when investigating immune responses, leading to conflicting results.

Several genetic studies highlight the relevance of the immune system in AD and PD. For example, single nucleotide polymorphisms (SNPs) in the HLA genetic loci have been associated with a protective effect for both diseases, and are primarily driven by polymorphisms present in most HLA-DRB1*04 subtypes [51–53]. In addition, genetic risk factor *APOEε4*, known to increase the presence of AD co-pathology [54], was found to be associated with a greater expression of microglial markers CD68 and HLA-DR and reduced expression of Iba1 [55]. Contrarily, mutations in the glucocerebrosidase (GBA) gene are associated with pathologically pure forms of DLB without AD co-pathology [56]. For that reason, we believe it is important to investigate the frequency of HLA-DRB1*04, *APOEε4* and *GBA1* and their effect on pathology and glial load in our cohort.

Our study aims to determine whether there is a different pattern of neuroinflammation in DLB cases with AD co-pathology compared to pure DLB and pure AD cases. In addition, we aim to investigate whether microglial and astroglial activation is associated with α-syn pathology or with AD co-pathology in mixed DLB + AD. Next, we will investigate whether microglial and astroglial activation is higher in regions with a higher burden of α-syn and concomitant p-tau and Aβ pathology. Lastly, we genotyped three common genetic risk genes in DLB and AD, i.e. *GBA1* mutation and frequency of *APOEε4* and HLA-DRB1*04 alleles, to investigate their effect on pathology burden and inflammatory response in our cohort [52, 56, 57]. To answer these questions, immunostaining, quantitative image analysis, confocal microscopy and genotyping were performed in limbic and cortical brain regions in a cohort of mixed DLB + AD, pure DLB, pure AD and controls.

## Methods

### Study cohort

For this study, a total of 77 brain donors were included. Postmortem brain tissue from pure AD (*n =* 16), pure DLB (*n =* 15), mixed DLB + AD (*n =* 35) and controls (*n =* 11) was obtained from the Netherlands Brain Bank (NBB; Amsterdam, the Netherlands; http://brainbank.nl), and the Normal Aging Brain Collection Amsterdam (NABCA; Amsterdam, the Netherlands; http://nabca.eu). Written informed consent for brain autopsy, the use of the material and the use of clinical information for research purposes was collected from all donors. All procedures of NBB and NABCA were approved by the local ethical board of VU University Medical Center, Amsterdam. For donor characteristics, see Supplement 1, Additional File 1.

Donors were selected based on clinical and neuropathological diagnosis. The inclusion criteria for a pure DLB diagnosis were (1) a clinical diagnosis of probable DLB according to the DLB consortium criteria [3], (2) the presence of diffuse-neocortical or limbic-transitional α-syn pathology at the time of autopsy (Braak α-syn stage ≥ 4), and 3), AD pathology was absent or low according to the National Institute of Aging-Alzheimer’s association (NIA-AA) guidelines [58]. Donors were included in the pure AD group if they met the following criteria: (1) a clinical diagnosis of probable AD and (2) intermediate or high levels of AD pathology [58]. Donors with an atypical clinical AD phenotype, as defined by Boon et al. [59], were excluded. The inclusion criteria for the mixed DLB + AD group were (1) a clinical diagnosis of probable DLB [3], (2) the presence of diffuse-neocortical or limbic-transitional α-syn pathology, and (3) moderate or high levels of AD pathology [58]. The control group was age-matched, and donors were included if there were no records of any neurological disorders. Only donors of which sufficient clinical information for classification and brain tissue samples were available, were included.

### Detection of pathology and inflammation with immunohistochemistry

Tissue blocks were collected from the following brain regions: the amygdala, hippocampus (HipMid) and middle temporal gyrus (MTG). Sections were cut at 6–7 μm thickness from formalin-fixed, paraffin-embedded blocks and mounted to glass slides. Immunohistochemistry (IHC) was performed with antibodies against full-length alpha-synuclein (1/500, *clone KM51, Monosan Xtra, The Netherlands*), amyloid-beta (1/500, *clone 70 6F/3D, Dako, Denmark*), and phosphorylated tau (1/500, *p-tau, clone AT8, Thermo Fisher Scientific, USA)* and microglial markers CD68 (1/500, *Mouse monoclonal, Clone KP1, Catalogue No. M0814, DAKO)*, HLA-DR (1/400, Mouse *monoclonal, Clone CR3/43, Catalogue No. M0775, DAKO*), and Iba1 (1/2000, *Goat polyclonal, Catalogue No. ab5076, Abcam)*, and astrocytic marker GFAP (1/1000, *Mouse Anti-GFAP, Catalogue No. G3893, SIGMA)*. See Supplement 2, Additional File 1 for details of the staining protocols.

### Microscopic analysis

**Fig. 1:**
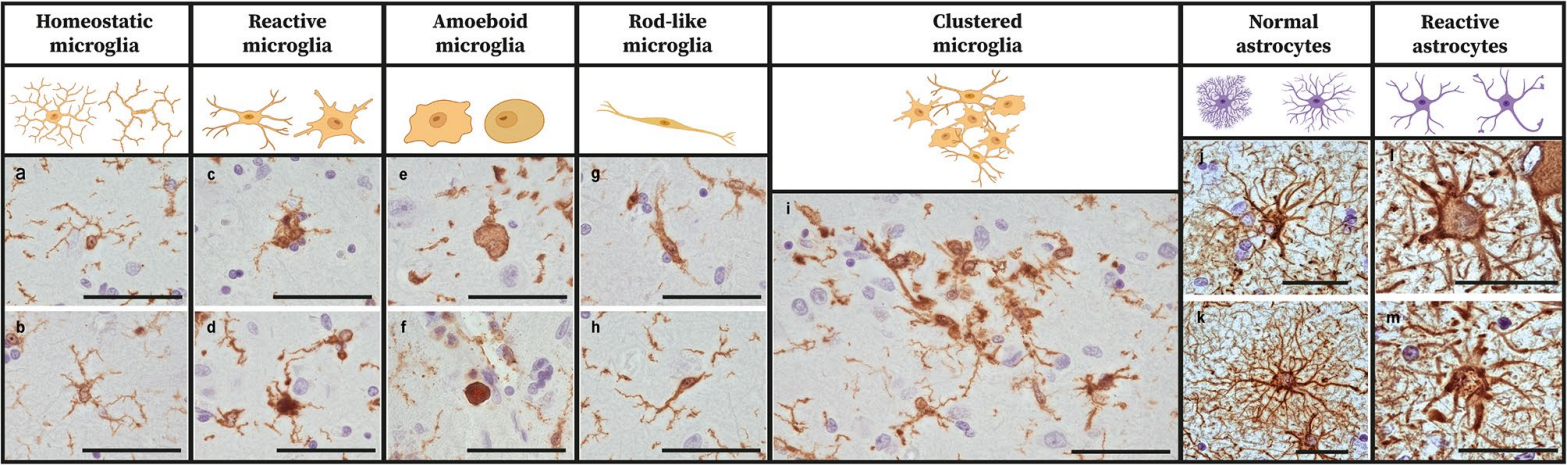
**The morphology of microglial and astrocytic structures in the amygdala.** Schematic drawings of the common morphological microglial and astrocytic structures including representative immunostained images of these morphological subtypes in the amygdala. Morphological nomenclature is stated as derived from the previous papers [64–67]. Immunostaining was performed with microglial marker Iba1 for images **a-i** and with astrocytic marker GFAP for images **j-m**. Homeostatic microglia have a small soma and long complex processes as shown in a control donor (**a)** and a pure DLB case (**b**). Reactive microglia show a larger soma with retracted and fewer processes as demonstrated in a mixed DLB + AD (**c**) and pure AD (**d**) case. Amoeboid microglia typically lack processes and have a large soma, here demonstrated in a pure AD (**e**) and control donor (**f**). Rod-like microglia are characterized by a long thin soma with long extended processes, shown here in a pure DLB case (**g**) and a control donor (**h**). Clustered microglia are characterized by the proximity of multiple microglial somas, and mostly consist of amoeboid and reactive microglial cells. An example of a large microglial cluster is shown in a pure AD case (**i**). Normal physiologic astrocytes have a small cell soma with long, thin and complex processes as shown in a pure DLB case (**j**) and a control donor (**k**). Reactive astrocytes have a larger cell soma with short, retracted and large processes as shown in a pure AD case (**l, m**). Scale bar represents 50 μm in each image

IHC sections were quantitatively assessed after digitization with the Vectra Polaris Quantitative Pathology Imaging System (PerkinElmer, USA) at 20x magnification. Representative areas [see Supplement 3, Additional File 1] were manually delineated using QuPath software, version 3.0.0 [60], in which the amygdala was delineated as entire amygdaloid complex [61], the hippocampus annotation was divided into the following subregions: dentate gyrus (DG), cornu ammonis (CA1, CA2, CA3, CA4), subiculum and parasubiculum [62], and the cortical regions (entorhinal cortex (EntC), parahippocampal gyrus (PHG), fusiform gyrus (FusG) and temporal cortex (TC)) had to include all six cortical layers within non-curved areas. All images were analyzed with QuPath open source software [60] using in-house developed scripts [see Supplement 4, Additional File 1], quantifying the percentage of total DAB-stained area, in this study referred to as immunopositivity [see Supplement 5, Additional File 1]. A Color Deconvolution Stains tool in QuPath was used to correct for unspecific background. Besides, images of microglial and astroglial morphology in the amygdala, CA1, CA2 and PHG were shown, based on microscopic assessment at 40x magnification of representative pure and mixed DLB cases. In addition, a semi-quantitative analysis on glial morphology in the amygdala was performed using QuPath software. A grid of 0.25 mm^2^ was placed within the area with the highest load of immunopositivity within the amygdala. For microglial morphology, five common microglial structures (homeostatic, reactive, amoeboid, rod-like and clustered microglia) were manually selected and counted within the 0.25 mm^2^ grid by QuPath software to calculate the density of the microglial subtype per area. To assess microglial morphology the Iba1 marker was selected, being a pan-marker that stains all microglial structures [63]. Astrocytic morphology was assessed semi-quantitatively in the same manner. Normal physiologic astrocytic structures, reactive astrocytic structures and total GFAP-positive cells were manually selected to calculate the density of the astrocytic subtypes per area. See [Fig. 1] for a schematic representation of the different glial structures, adjusted on the nomenclatures presented in previous papers [64–67].

### Multi-labeling immunofluorescence and confocal microscopy

To describe the morphology and co-localization of pathology and microglia, multi-labeling immunofluorescence staining in combination with 3D confocal laser scanning microscopy (CLSM) of microglia (Iba1), phosphorylated α-syn- (p-syn), Aβ- and p-tau-pathology was performed on a Leica TCS SP8 (Leica, Microsystems, Germany). Plaques and their co-localization with microglia were described in four mixed DLB + AD cases in the hippocampus. Images were deconvoluted using standard CLSM algorithms from Huygens Professional (Scientific Volume Imaging; Huygens, The Netherlands; https://svi.nl/Huygens-Professional), and their maximum projections were used to graphically represent structures of interest in ImageJ (ImageJ Fiji, National Institute of Health USA; https://imagej.nih.gov/ij/). Details of the protocol were described in Supplement 2, Additional File 1.

### *APOEε4*, HLA-DRB1*04 and *GBA1* genotyping

The frequencies of *APOEε4* and HLA-DRB1*04 subtype alleles and *GBA1* mutations were determined by genotyping using either the Infinium® NeuroChip Consortium Array v1.1 (Illumina) or Sanger sequencing (Invitrogen Life Technologies, Carlsbad, CA, USA). Details of the NeuroChip and Sanger sequencing methods used were described in previously published studies by Tunold et al. [68] and Moors et al. [69], respectively. HLA-DRB1*04 alleles were imputed from genotypes using the SNP2HLA software package with publicly available reference data from the Type 1 Diabetes Genetics Consortium [70]. The primer sequences are available upon request.

### Statistical analysis

All the statistical analyses were performed in IBM SPSS Statistics version 28. Demographics between disease groups were compared using a one-way ANOVA with post-hoc t-tests for continuous variables and a Fisher’s exact test with post-hoc pairwise comparisons for categorical variables. A linear mixed model analysis adjusted for age at death and sex was performed to estimate differences in glial morphological density of Iba1 and GFAP and to estimate immunoreactivity of each IHC marker (α-syn, Aβ, p-tau, Iba1, HLA-DR, CD68 and GFAP) between disease groups within the different regions and between different genotypes. To estimate differences in immunoreactivity between brain regions over all groups, a correction for disease group was added to the analysis. Associations between the various neuroinflammatory markers (Iba1, HLA-DR, CD68 and GFAP) and pathology markers (α-syn, Aβ and p-tau) were analyzed using a linear mixed model correcting for age at death and sex. Pathology or neuroinflammatory marker analysis was further adjusted for α-syn load, Aβ load or p-tau load. Based on the results of the linear mixed model analysis, standardized regression coefficients (r) were calculated for the four diagnostic groups. Significance for all analyses was based on p-values < 0.05 after FDR-correction for multiple comparisons, with subsequent reporting of FDR-corrected p-values [71]. Heatmaps and graphs were generated using GraphPad Prism version 9.3.1 (GraphPad Software, San Diego, California, USA).

## Results

### Demographics of the cohort

A shorter survival time between disease diagnosis and death was observed in mixed DLB + AD (6 ± 3 years) than in pure DLB (8 ± 3 years; *p* = 0.030) and pure AD (10 ± 5 years; *p* < 0.001) cases [Table 1]. Higher Braak stages for neurofibrillary tangles (NFT) (*p* = 0.003), Thal stages for Aβ (*p* < 0.001) and Consortium to Establish a Registry for Alzheimer’s Disease (CERAD) scores (*p* < 0.001) were observed in pure AD when compared to mixed DLB + AD cases. No significant difference in Braak stage for α-syn was observed between pure DLB and mixed DLB + AD cases. Heterozygous and homozygous *APOEε4* were more frequent in mixed DLB + AD (9% non-carriers, 79% heterozygous and 12% homozygous) than in pure DLB (62% non-carriers, 38% heterozygous and 0% homozygous; *p* < 0.001) and controls (73% non-carriers, 27% heterozygous and 0% homozygous; *p* < 0.001), but did not significantly differ with pure AD cases (31% non-carriers, 56% heterozygous and 13% homozygous). A higher frequency of *GBA1* mutations, varying in risk variant [see Table 1**]**, was observed in pure DLB cases (62%) compared to mixed DLB + AD cases (12%) (*p* < 0.001). There were no significant differences in the frequency of HLA-DRB1*04 alleles between diagnostic groups.

**Table 1:**
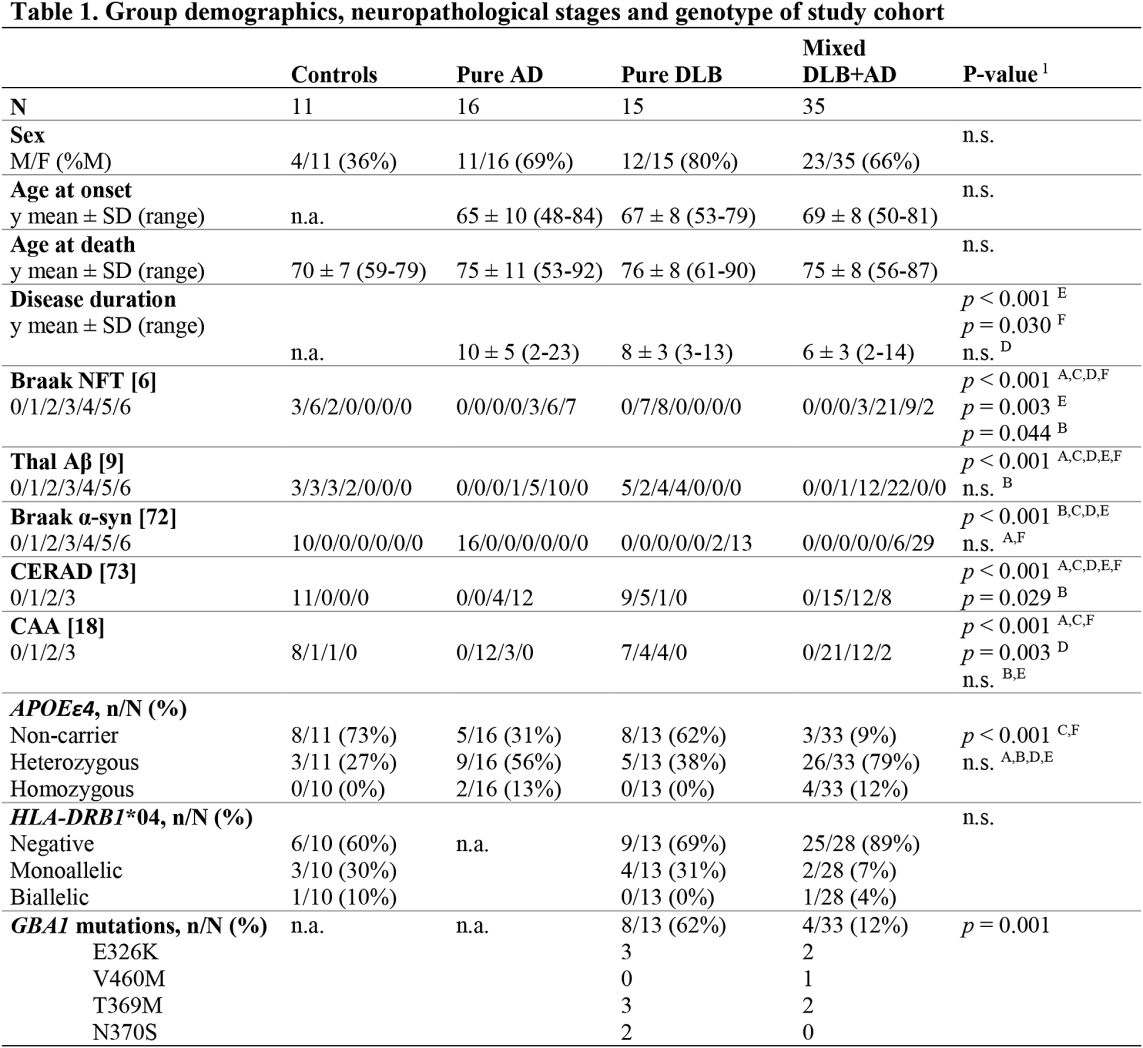
Group demographics, neuropathological stages and genotype of study cohort

N.a. = not available. N.s. = no significant difference between groups. A = controls vs. pure AD; B = controls vs. pure DLB; C = controls vs. mixed DLB + AD; D = pure AD vs. pure DLB; E = pure AD vs. mixed DLB + AD; F = pure DLB vs. mixed DLB + AD. CAA = cerebral amyloid angiopathy; CERAD = Consortium to Establish a Registry for Alzheimer Disease; *APOEε* = apolipoprotein E; HLA-DRB1*04 = human leukocyte antigen DRB1*04 subtypes; *GBA1* = glucocerebrosidase gene 1. Groups were compared using a one-way ANOVA with post-hoc t-tests for continuous and a Fisher’s exact test with post-hoc pairwise comparisons for categorical variables. Post-hoc tests were performed if overall test was significant

### Regional differences in pathological load across diagnostic groups

**Fig. 2:**
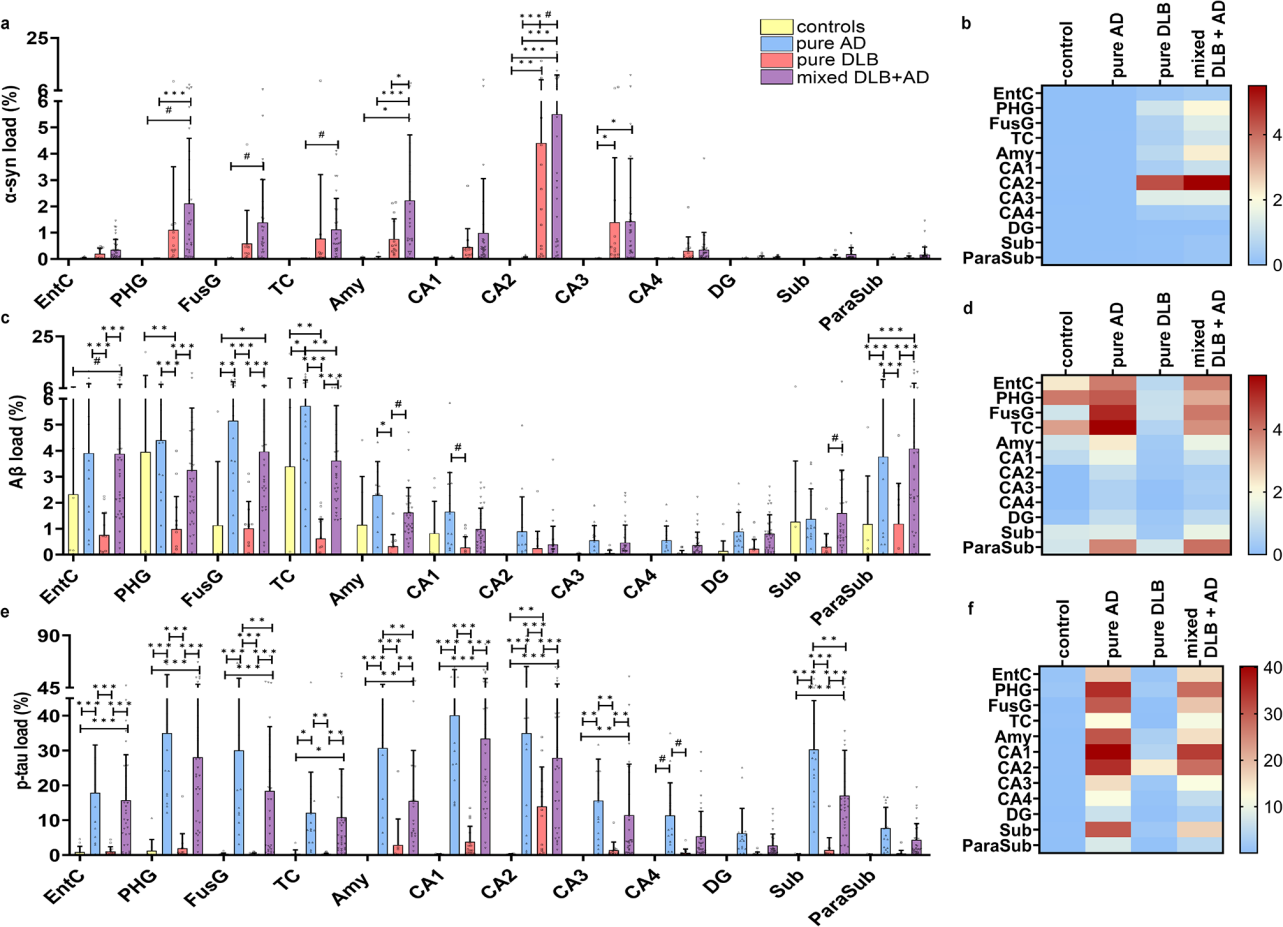
**Distribution patterns of pathology in limbic and neocortical brain regions across diagnostic groups.** Distribution of α-syn, Aβ and p-tau pathology is visualized in a scatter plot with box and heatmaps of mean pathological load [SD] in which the color-coded legend on the right indicates the %area of pathology. **a-c** α-Syn load was higher in the amygdala of mixed DLB + AD cases than in pure DLB cases. **d-f** Aβ pathology was higher in the TC of pure AD than mixed DLB + AD cases. **g-i** p-Tau was higher in the FusG, amygdala and subiculum of pure AD than mixed DLB + AD cases. A linear mixed model analysis with correction for age at death and sex was performed to estimate group differences. * *p* < 0.05, ** *p* < 0.01, *** *p* < 0.001, # *p* < 0.05 without significance after correction for multiple comparisons. EntC = entorhinal cortex; PHG = parahippocampal gyrus; FusG = fusiform gyrus; TC = temporal cortex; Amy = amygdala; CA = cornu ammonis; DG = dentate gyrus; Sub = subiculum; ParaSub = parasubiculum

For all cases, the pathological loads of α-syn, Aβ and p-tau were assessed in cortical regions (EntC, PHG, FusG and TC), the amygdala and hippocampus (CA1-4, DG, subiculum and parasubiculum) [Fig. 2]. Mixed DLB + AD cases had a higher α-syn load in the amygdala (*p* = 0.017) compared to pure DLB cases. The Aβ load was similar in mixed DLB + AD and pure AD cases, and was only higher in the TC of pure AD cases (*p* = 0.001). Higher p-tau loads were found for pure AD cases when compared to mixed DLB + AD cases in the amygdala, subiculum and FusG (*p* = 0.002, *p* = 0.001, *p* = 0.004, respectively), and did not differ in other regions. By design, mixed DLB + AD cases had higher loads of AD-pathology than pure DLB cases and higher loads of α-syn than pure AD cases [see comparisons in Supplement 6, Additional File 1]. Across groups, the highest α-syn load existed in the CA2, and was significantly higher than in all the other brain regions examined (*p* < 0.001).,The highest Aβ load was found in cortical regions and was significantly higher in the EntC, PHG, FusG and TC than in the DG, CA1-4, subiculum and amygdala (all *p* < 0.001). Similar to the α-syn load, the highest p-tau load was found in the CA1 and CA2 and was higher when compared to the DG, CA3, CA4, amygdala, subiculum, parasubiculum, EntC, FusG and TC (all *p* < 0.001). Additional regional differences in α-syn, Aβ and p-tau loads are stated in Supplement 7, Additional File 1.

### Mainly amoeboid and reactive microglial structures and physiologic astrocytes in the amygdala of mixed DLB cases

As previously described, the amygdala is a region suggested to be prone to start pathological misfolding of α-syn, Aβ and p-tau pathology [11, 12]. In addition, after the CA1 and CA2 region, where mostly neurites and a few plaques were observed, the highest α-syn, p-tau and microglial load and a wide variety of pathological structures existed in the amygdala in our cohort [see Supplement 7, Additional File 1]. Therefore, we decided to describe and count microglial and astrocytic structures in the amygdala for all groups [see Fig. 1 + 3]. Group differences in the density of different morphological structures were estimated with a linear mixed model analysis with correction for age at death and sex. The density of homeostatic microglia, characterized by a small soma and long complex processes, was significantly higher in controls than in mixed DLB + AD, pure AD (*p* < 0.001 in both) and pure DLB (*p* = 0.01), and in pure DLB than in mixed DLB + AD and pure AD cases (*p* < 0.001). In contrast, the density of reactive microglia, characterized by a large soma and short retracted processes, was higher in pure AD than in pure DLB (*p* = 0.002) and controls (*p* < 0.001) and in mixed DLB + AD than in pure DLB and controls (*p* < 0.001 in both). Likewise, the density of amoeboid microglia, characterized by a large round soma without processes, was higher in pure AD than in pure DLB, controls (*p* < 0.001 in both) and mixed DLB + AD (*p* 0.002), and in mixed DLB + AD than in pure DLB (*p* = 0.005) and controls (*p* = 0.026). No differences in rod-like microglia, characterized by a long, thin soma and long but simple processes, existed between groups. The density of clustered microglia in the amygdala was higher in pure AD cases than in all other groups (*p* < 0.001 in all). The density of normal physiologic astrocytes, characterized by a small soma and long complex processes, was higher in mixed DLB + AD than in pure AD (*p* < 0.001) and controls (*p* = 0.002), and in pure DLB than in pure AD (*p* = 0.012). In contrast, the density of reactive astrocytes, characterized by a large soma with short retracted processes, was higher in pure AD cases than in all other groups (*p* < 0.001 in all). Total GFAP-positive astrocytic density, which is suggested to characterize astrocytic reactivity [66], was higher in pure AD than in pure DLB cases (*p* = 0.003) or controls (*p* < 0.001).

**Fig. 3:**
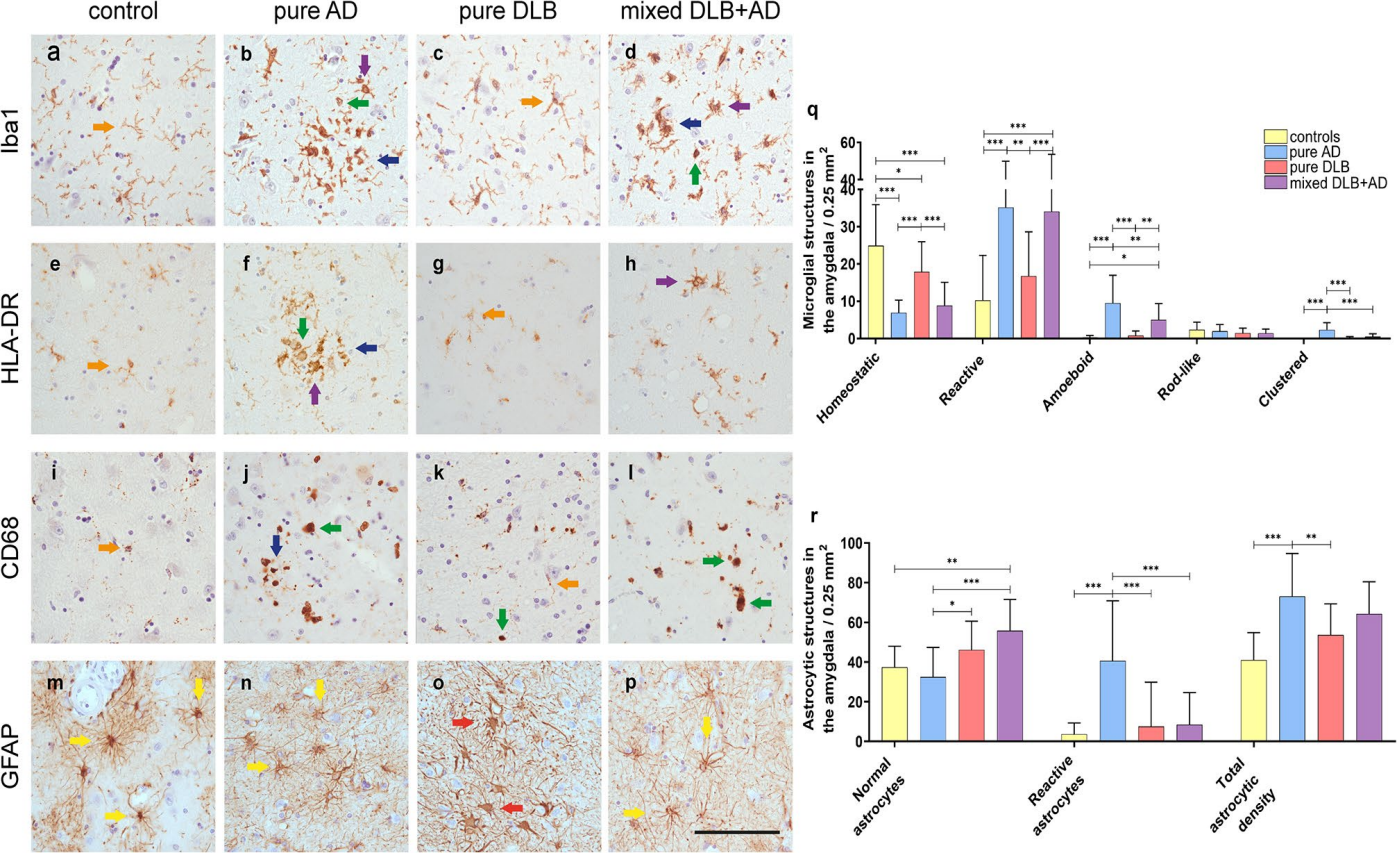
**Amoeboid and reactive microglial morphology and physiologic astrocytic morphology in mixed DLB cases.** Representative images of microglial (**a-l**) and astrocytic (**m-p)** morphology in the amygdala with a scatter plot of the mean density of microglial Iba1-positive (**q**) and astrocytic (**r)** structures in the amygdala [SD]. **a, e, i** In control cases, homeostatic microglia with long processes (orange arrowhead) were observed. **b, f, j** In pure AD cases, amoeboid microglia with a large and round soma (green arrowhead), reactive microglia with short retracted processes (purple arrowhead) and clustered microglia (blue arrowhead) were observed. **c, g, k** In pure DLB cases, homeostatic (orange arrowhead) microglia and small amoeboid microglia (green arrowhead) were observed. **d, h, l** Mixed DLB + AD cases demonstrated amoeboid (green arrowhead), reactive (purple arrowhead) and clustered microglia (blue arrowhead). **m, n, p** In control, pure DLB and mixed DLB + AD cases normal physiologic astrocytes with a small soma and long, thin, complex processes (yellow arrowhead) were observed. **o** A higher total astrocytic density and reactive astrocytes with a swollen soma and short retracted processes (red arrowhead) were observed in pure AD cases. **q** The density of homeostatic microglia in the amygdala was significantly higher in control and pure DLB cases than in mixed DLB + AD and pure AD cases. The density of reactive and amoeboid microglia in the amygdala was higher in mixed DLB + AD and pure AD cases than in pure DLB and control cases. The density of clustered microglia in the amygdala was higher in pure AD cases than in all other groups. **r** The density of normal astrocytes was higher in mixed DLB + AD and pure DLB cases than in pure AD and controls, while the density of reactive astrocytes was higher in pure AD cases than in all other groups in the amygdala. Total astrocytic density in the amygdala was higher in pure AD than in pure DLB cases or controls. Scale bar in **p** is identical for all images and represents 100 μm

Second, differences in glial morphology were described for the amygdala, CA1 and PHG in mixed DLB + AD and pure DLB cases [see Supplement 8, Additional File 1]. Only small differences in microglial structures between regions were observed, but in limbic regions, particularly the amygdala, microglia seemed to be more often in a morphologically reactive state. Astrocytes in the PHG and CA1 seemed to have a larger soma with shorter processes, while astrocytes in the amygdala had a smaller soma with long processes. However, it is important to note that a semi-quantitative assessment was only performed for the amygdala.

### Microglial response in mixed DLB is more severe than in pure DLB

**Fig. 4:**
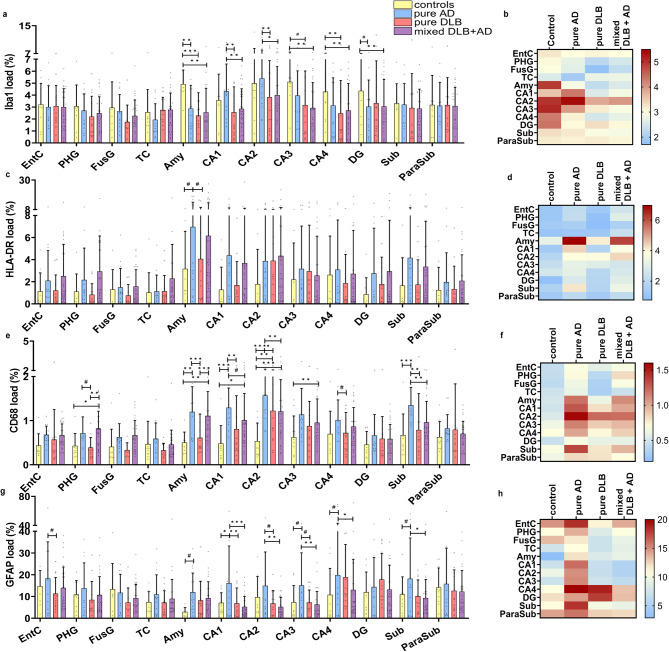
**Microglial response was more severe in mixed DLB cases, while astrocytic response was similar to pure DLB.** Distribution patterns in regions of interest are visualized in a scatter plot with box and heatmaps of mean microglial load [SD], in which the color-coded legend on the right indicates the %area of immunopositivity. **a-c** Iba1-positive microglial load was higher in controls than in pure AD in the amygdala, than in pure DLB in the amygdala and CA4, and higher than in mixed DLB + AD in the amygdala, CA3, CA4 and DG. **d-f** The highest reactive microglial load, measured by HLA-DR, was observed in the amygdala of mixed DLB + AD and pure AD cases, but was not significantly higher after correction for multiple comparisons. **g-i** Mixed DLB + AD showed a higher reactive microglial load, as measured by CD68, than pure DLB in the PHG and amygdala. Pure AD had a higher CD68 positive microglial load than mixed DLB + AD in the CA2 and subiculum. **j-l** Pure AD cases had a higher astrocytic load in the CA1, CA2, CA3, CA4 and subiculum than mixed DLB + AD cases. A linear mixed model analysis with correction for age at death and sex was performed to estimate group differences. * *p* < 0.05, ** *p* < 0.01, *** *p* < 0.001, # *p* < 0.05 without significance after correction for multiple comparisons. EntC = entorhinal cortex; PHG = parahippocampal gyrus; FusG = fusiform gyrus; TC = temporal cortex; Amy = amygdala; CA = cornu ammonis; DG = dentate gyrus; Sub = subiculum; ParaSub = parasubiculum

Iba1 is located at the outer membrane and is a pan-marker that stains all present microglia including their processes [63], and will be referred to as ‘total microglia’ throughout this paper. CD68 is mostly expressed by lysosomes and demonstrates round microglial structures without processes, while HLA-DR is located at the cytoplasm. Both CD68 and HLA-DR are markers that are mostly upregulated in phagocytosing and thus reactive microglia and foamy macrophages [74], and will be referred to as ‘reactive microglia’. However, it should be noted that not only reactive, but also homeostatic microglia will be stained by CD68 and HLA-DR. Total microglial (Iba1-positive) and reactive microglial (CD68- and HLA-DR-positive) loads were quantified within the amygdala, hippocampal subregions and cortical regions [Fig. 4]. The total microglial load (Iba1-positive), observed in the control group, was higher in the amygdala when compared to pure AD, pure DLB and mixed DLB + AD (*p* = 0.016, *p* < 0.001, *p* = 0.002, respectively), in the DG, CA3 and CA4 when compared to mixed DLB + AD (*p* = 0.045, *p* = 0.012, *p* = 0.015, respectively) and in the CA4 when compared to pure DLB (*p* = 0.012). Pure AD cases showed a higher load of total microglia in the CA1 and CA2 when compared to pure DLB cases (*p* = 0.012, *p* = 0.042) and to mixed DLB + AD cases (*p* = 0.021, *p* = 0.027). No differences in total microglial load were observed between mixed and pure DLB cases. Across all groups, the highest total microglial load was found in the CA2, which was significantly higher than in all the other brain regions assessed (*p* < 0.001).

Second, reactive microglia were examined [63, 74]. In [Fig. 4c-d], mixed DLB + AD and pure AD cases appeared to have a higher reactive microglial load (HLA-DR-positive) in the amygdala, CA1 and subiculum than pure DLB cases. However, after correction for multiple comparisons, no statistical significant differences in reactive microglial loads (HLA-DR-positive) were observed between groups. We then analyzed the reactive microglial load (HLA-DR-positive) between regions and found that it was the highest in the amygdala when compared to all other regions (all *p* < 0.001). As shown in [Fig. 4e-f], a high reactive microglial load (CD68-positive) was observed in limbic regions of mixed DLB + AD and pure AD cases. Reactive microglial load (CD68-positive) was significantly higher in mixed DLB + AD cases compared to pure DLB cases in the amygdala and PHG (*p* < 0.001 and *p* = 0.004 respectively), in pure AD cases when compared to pure DLB cases in the CA1, CA2, the subiculum and amygdala (*p* = 0.004, *p* = 0.028, *p* = 0.002, *p* < 0.001, respectively) and in pure AD cases when compared to mixed DLB + AD cases in the CA2 and subiculum (*p* = 0.01 and *p* = 0.014 respectively). The highest reactive microglial load (CD68-positive) was found in the CA2 and was higher than in all other regions (all *p* < 0.001).

### Astrocytic response in mixed and pure DLB cases is similar

GFAP is a marker that becomes upregulated in reactive astrocytes [75] and was quantified within the amygdala, hippocampal subregions and cortical regions [Fig. 4g-h]. A significantly higher astrocytic load (GFAP-positive) was observed in pure AD cases than in mixed DLB + AD in the CA1, CA2, CA3, CA4 and subiculum (*p* < 0.001, *p* = 0.003, *p* = 0.01, *p* = 0.042, *p* = 0.006 respectively) and in pure DLB in the CA1 (*p* = 0.014). No differences in astrocytic load were observed between mixed and pure DLB cases. In contrast with the abundance of α-syn and p-tau pathology and microglial load, we observed the lowest astrocytic load in the amygdala, CA1 and CA2 when compared to other regions. The highest astrocytic load was found in the CA4 compared to the PHG, FusG, TC, amygdala, CA1, CA2, CA3 (*p* < 0.001 in all) and subiculum (*p* = 0.002). Additional regional differences in Iba1, HLA-DR, CD68 and GFAP loads are shown in Supplement 7, Additional File 1.

### Microglial response in close proximity to AD plaques in mixed DLB cases

**Fig. 5:**
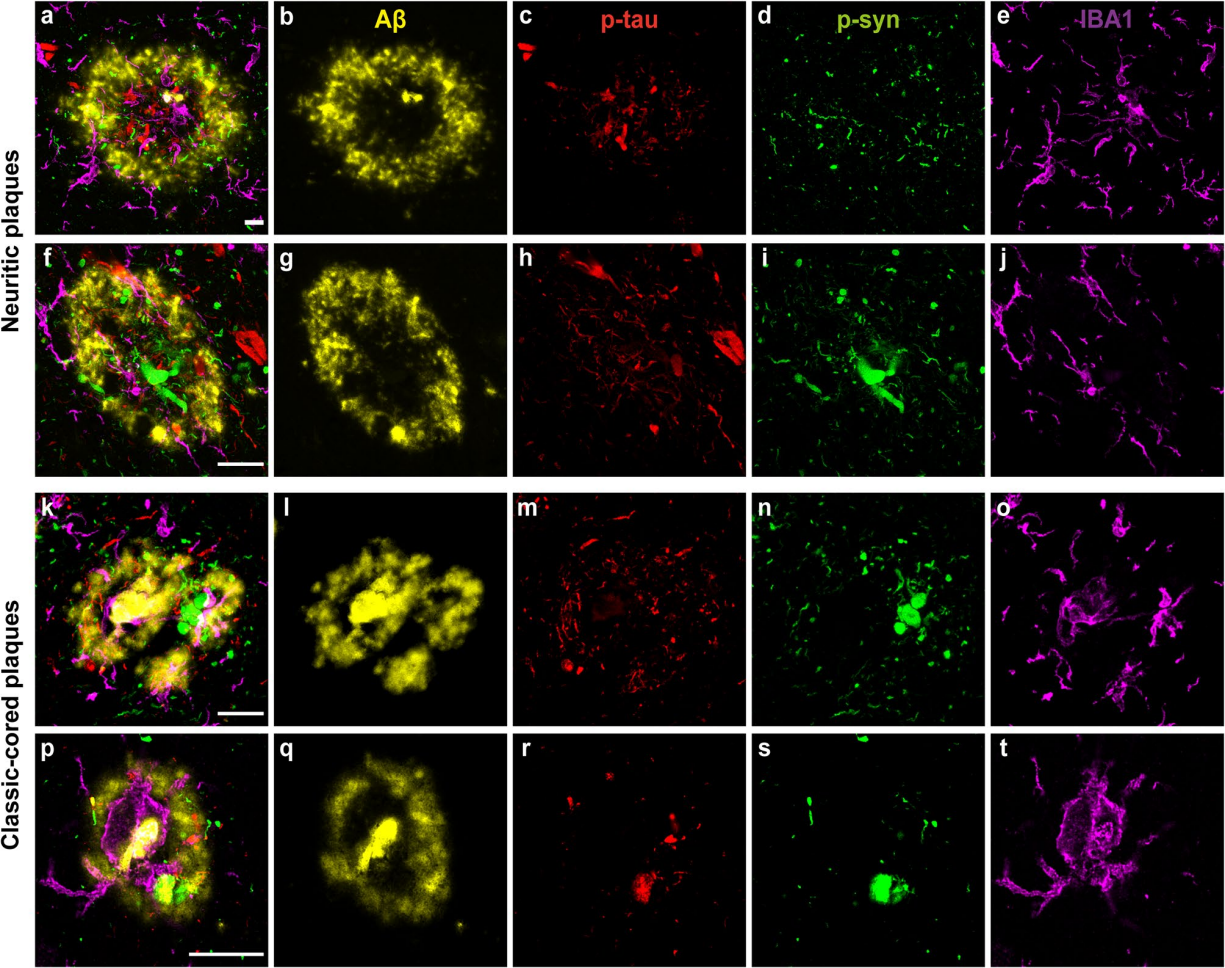
**Representative confocal microscopy images of microglia in close proximity to AD-plaques in mixed DLB cases.** Localization of pSer129-syn (green), Aβ (yellow), p-tau (red) and Iba1+ (purple) microglia in neuritic and classic cored plaques in the CA1, EntC and TC regions demonstrated with confocal microscopy. **a-e** Many large clustered and amoeboid Iba1-positive microglia with short, thick processes in the neuritic plaque in the CA1 within and in closer proximity to neuritic p-tau and p-syn accumulation. **f-j** Another example of rod-like and amoeboid Iba1-positive microglia in a neuritic plaque in the CA1. **k-o** Amoeboid and clustered microglia within and surrounding a classic-cored plaque in the EntC with neuritic p-tau and p-syn pathology. **p-t** Reactive microglial cell with large cell soma and thick processes within a classic-cored plaque with mainly Aβ pathology present in the TC. The scale bars in **a, f, k, p** are identical for all images in one row and represent 20 μm in all

When we studied the spatial relationship between microglia and plaques with multi-labeled immunofluorescence for Iba1, Aβ, p-tau and p-syn staining (pSer129), many large clustered and amoeboid Iba1-positive microglia were observed in and surrounding neuritic plaques consisting of Aβ and p-tau pathology [Fig. 5a, f, k]. We showed neuritic and classical dense-cored plaques as previously described by Walker et al., derived from the preceding description by Thal et al. [76, 77]. One image showed a large amoeboid Iba1-positive microglial cell with a large cell soma completely within a classic cored plaque that mainly consisted of Aβ and p-tau pathology [see Fig. 5p-t]. Direct co-localization between LBs or LNs and Iba1-positive microglia was not found. However, punctate p-syn, which can be either astrocytic, neuritic or synaptic α-syn, was present in the AD-plaques.

### Whereas microglia associated with AD-, astroglia associated with amygdalar α-syn-pathology in mixed DLB

Standardized regression coefficients between microglial and astroglial, and α-syn, Aβ and p-tau load were assessed for each group over all regions, with adjustments for age at death, sex and α-syn, Aβ and p-tau loads [see Table 2]. The α-syn load in pure AD cases and controls was negligible, hence these associations were not described. Total microglial load (Iba1-positive), associated negatively with Aβ load in pure AD and mixed DLB + AD (*r* = -0.40, *p* < 0.001; *r* = -0.11, *p* = 0.012) and positively with p-tau load in pure AD (*r* = 0.18, *p* = 0.021). Reactive microglial load (HLA-DR-positive) correlated negatively with Aβ load in pure AD, pure DLB and mixed DLB + AD (*r* = -0.13, *p* = 0.033; *r* = -0.16, *p* = 0.048; *r* = -0.15, *p* < 0.001) and positively with p-tau load in pure AD, pure DLB and mixed DLB + AD (*r* = 0.24, *r* = 0.28 and *r* = 0.12, *p* < 0.001). Reactive microglial load (CD68-positive), again associated negatively with Aβ load in pure AD and mixed DLB + AD (*r* = -0.36 and *r* = -0.21, *p* < 0.001) and positively with p-tau load in pure AD, pure DLB, and mixed DLB + AD (*r* = 0.38, *r* = 0.30 and *r* = 0.28, *p* < 0.001). Astrocytic load was not associated with pathological load in the assessed diagnostic groups.

**Table 2:**
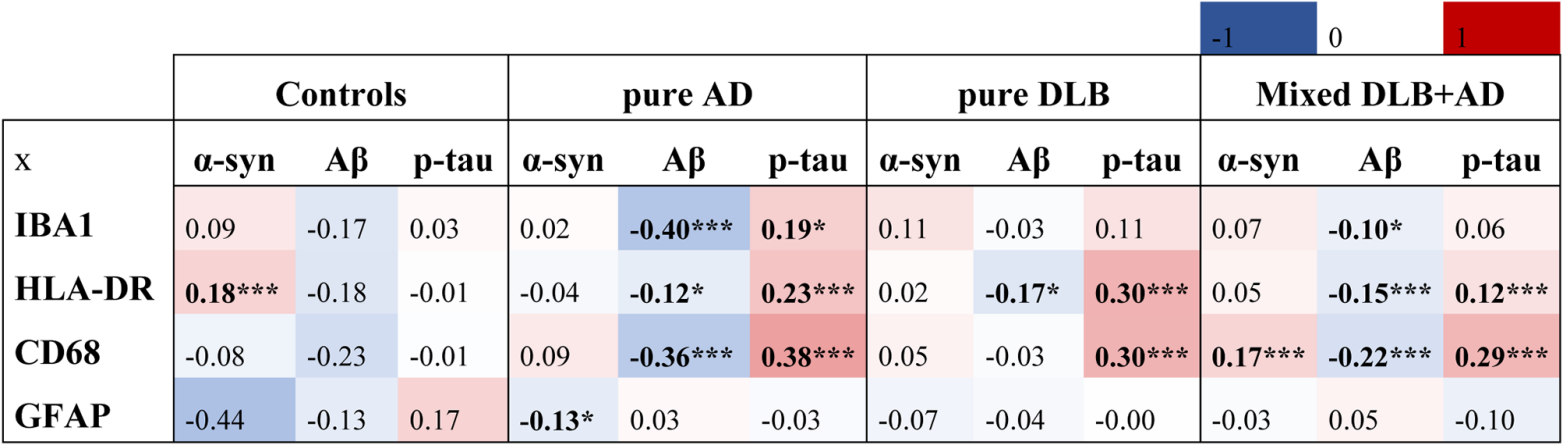
Strong associations between microglial load and AD pathology existed in disease groups

Heatmap of standardized regression coefficients between microglial or astrocytic load and α-syn, Aβ and p-tau pathological load over all groups. Positive standardized regression coefficients are depicted in red, while negative standardized regression coefficients are denoted in blue in the heatmap. Significance is demonstrated in bold as * *p* < 0.05, ** *p* < 0.01, and *** *p* < 0.001

We then examined associations between microglial and astroglial and α-syn, Aβ and p-tau load within each region in mixed DLB + AD cases to see whether regional differences could be highlighted [see Supplement 9, Additional File 1]. Strong associations between reactive microglial load (CD68-positive), and p-tau load were observed in the PHG and TC (*r* = 0.41, *p* = 0.048; *r* = 0.72, *p* < 0.001). Reactive HLA-DR-positive microglial load associated positively with the α-syn load in the CA1 (*r* = 0.53, *p* = 0.024). Interestingly, astrocytic load showed a strong association with α-syn load in the amygdala (*r* = 0.69, *p* < 0.001).

### More AD-pathology and microglial response in *APOEε4* carriers and less AD-pathology in *GBA1* carriers

**Fig. 6:**
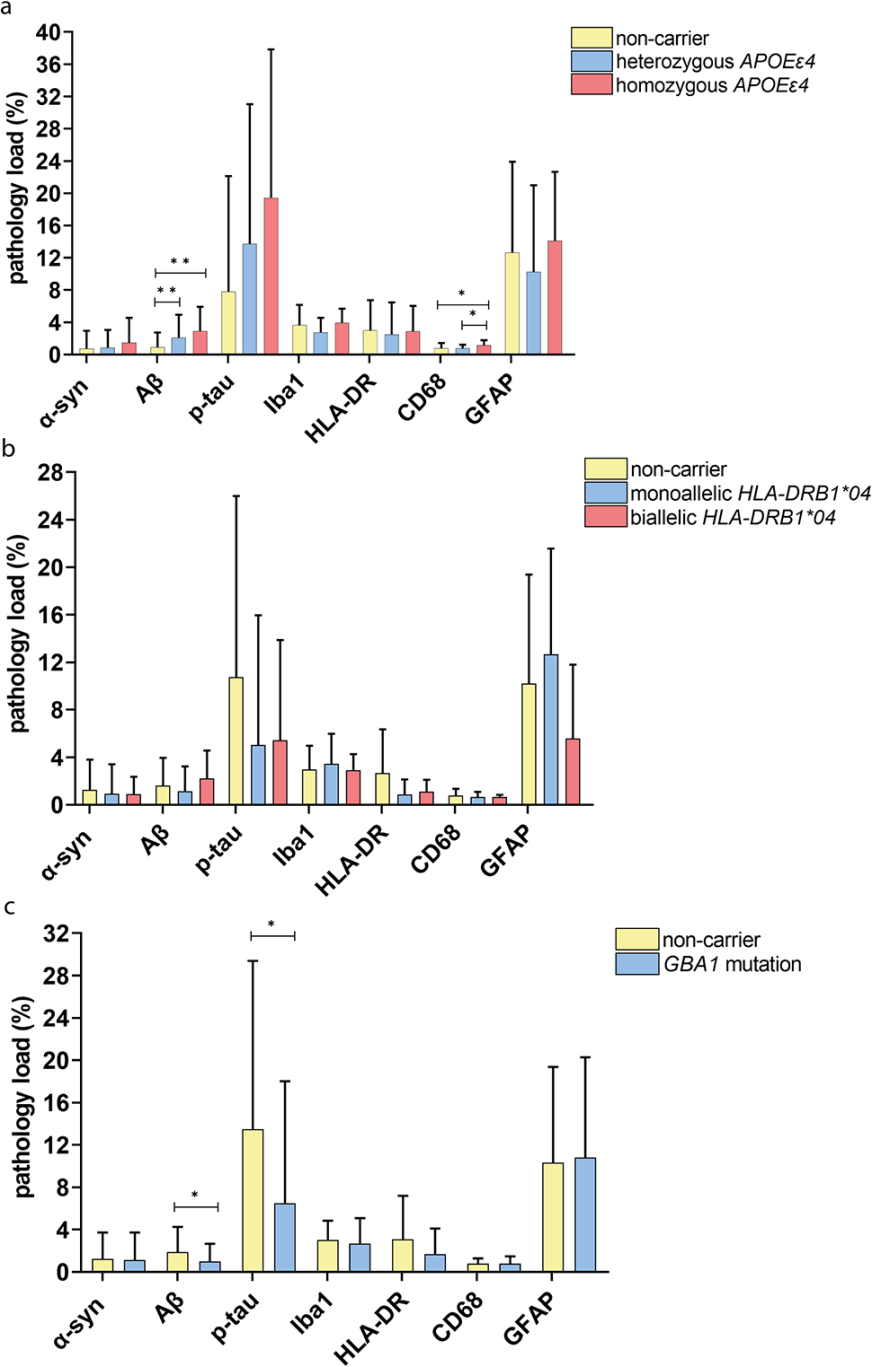
**Variants of *APOEε4 *and *GBA1 *genotype influenced the level of Aβ and p-tau pathology.** Immunopositivity of α-syn, Aβ, p-tau, Iba1, HLA-DR, CD68 and GFAP is visualized in a scatter plot with box of mean pathology load over all measured brain regions [SD]. **a** Aβ load increased with the number of *APOEε4* alleles and was significantly higher in heterozygous and homozygous than non-carriers. Microglial load was higher in homozygous than in heterozygous *APOEε4* or than in non-carriers. **b** No differences in pathology or micro- or astroglial load were observed between different frequencies of HLA-DRB1*04 alleles. **c** Pathogenic *GBA1* carriers had a higher Aβ and p-tau load than non-carriers. A linear mixed model analysis with correction for age of death and gender was performed to compare genotypes. * *p* < 0.05, ** *p* < 0.01, *** *p* < 0.001

Stratification of the frequency of *APOEε4* and HLA-DRB1*04 alleles and the presence or absence of pathogenic *GBA1* genotypes was performed to examine differences in microglial, astroglial and pathological loads between genetic variants [see Fig. 6]. The observed Aβ load was higher in heterozygous (*p* = 0.006) and homozygous (*p* = 0.006) *APOEε4* carriers than in non-carriers. In contrast, pathogenic *GBA1* mutation carriers had lower loads of Aβ and p-tau pathology than non-carriers did (*p* = 0.028, *p* = 0.030). Interestingly, homozygous *APOEε4* carriers had a higher reactive microglial load (CD68-positive) than heterozygous *APOEε4* (*p* = 0.015) or non-carriers (*p* = 0.036). No significant differences in microglial, astroglial or pathological load were observed between the other genetic variants.

## Discussion

Microglial reactivity in DLB associated strongly with Aβ and p-tau loads, while no association with astrocytic response was observed. Morphologically, amoeboid and reactive microglia were abundant in mixed DLB + AD and pure AD cases, while pure DLB and control cases mainly revealed homeostatic microglia and small astrocytes with thin processes. Reactive microglial load was higher in mixed DLB + AD compared to pure DLB cases, but DLB phenotypes, i.e. pure and mixed DLB cases, did not differ in astrocytic load. Finally, the highest microglial activity was found in the CA2 and amygdala, where concomitant α-syn and p-tau pathology also showed the highest loads.

Although we observed higher α-syn loads both in cortical regions and the amygdala in mixed DLB + AD cases than in pure DLB cases, which is consistent with previous studies [78–80], significance was only reached in the amygdala in our cohort. These findings suggest that the mixed DLB + AD cases are in a more advanced pathological stage of disease, according to the pattern of α-syn pathology distribution by Braak et al. [81]. However, DLB phenotypes were scored as Braak α-syn stage 5 or 6 and were not significantly different. This may be explained by the fact that Braak staging does not require evaluation of lesional density and assesses the presence of LBs and LNs [81], whereas we assessed a quantitative measure of total α-syn pathology. We showed that α-syn load was associated with reactive CD68-positive microglial load only in mixed DLB + AD cases. While several cell-culture and animal studies demonstrated a direct link between α-syn and microglial activation [37], few post-mortem studies were able to show this association in the cingulate cortex of PD [27], and in the transentorhinal cortex of pure and mixed DLB cases [29]. Furthermore, double-staining of HLA-DR microglia and α-syn only showed an association with 20% of the LBs in the cingulate cortex of PD [27], and it has been suggested that LBs alone are not sufficient to activate microglia [37]. Moreover, α-syn load strongly associated with astrocytic response in the amygdala in our cohort, a region where astrocytic α-syn was previously reported to be predominant [11]. In addition, the presence of astrocytic α-syn in the amygdala and other brain regions has recently been demonstrated by studying various post-translational modifications (PTMs) of α-syn [43]. These observations suggest that reactive microglia and astrocytes might partly respond to the increase of α-syn pathology in mixed DLB + AD cases, or contribute to a more aggressive synucleinopathy.

As previously described and confirmed by our study, early affected regions in AD exhibited similar Aβ- and p-tau- pathological loads in pure AD and mixed DLB + AD cases [4], while regions affected in more advanced disease stages [6], such as limbic regions for p-tau and cortical regions for Aβ pathology, were more heavily affected in pure AD cases [4, 82]. The presence of p-tau did show a positive association with reactive microglial load for pure AD, pure DLB and mixed DLB + AD. Likewise, a recent study using 3D confocal microscopy demonstrated that p-tau was associated with microglial morphological features in the hippocampus of DLB and AD donors [21]. Interestingly, we observed an overall negative association between Aβ pathology (including diffuse plaques) and microglial load in pure AD and mixed DLB + AD cases. Previous studies have indicated that microglia are able to engulf extracellular fibrillary Aβ plaques [83]; therefore, a negative association was expected. However, we observed that the association between Aβ pathology and reactive microglial load varied in different brain regions within mixed DLB + AD cases. A negative association was observed in the cornu ammonis, a region with a low Aβ load, while the association was reversed in cortical regions. Early microglial activation in AD has been shown to play a role in the phagocytosis of fibrillary Aβ plaques and precedes a pro-inflammatory harmful state of microglia in later disease stages [84]. These findings suggest that reactive microglia are able to successfully engulf and clear fibrillary Aβ plaques in regions that are affected only in later disease stages [84], such as the cornu ammonis, and that an overload of unengaged fibrillary Aβ plaques is observed in regions where the accumulation of Aβ plaques begins in early disease stages. The co-localization of microglia and Aβ plaques has been previously studied by Boon et al., in which the direct co-localization of clustered microglia in classic-cored plaques in AD was demonstrated via immunofluorescence multi-labeling staining [59]. Similarly, via detailed CLSM, we observed that Aβ plaques co-localize directly with reactive amoeboid microglia in limbic and cortical regions [Fig. 5.].

Previous studies likewise reported that Iba1-positive microglia were highly present in control cases and represented mainly homeostatic microglia, and when double-labeling IHC was performed, mainly reactive swollen amoeboid Iba1-positive microglia were CD68-positive [85]. In addition, previous studies have described the appearance of Iba1-positive microglia in the healthy aging brain as unchanged [30, 32] or even more prevalent than in DLB or AD [31, 63, 86]. However, the significance of the increase in total microglial load with age in the CNS is still unclear [87]. Presumably, this is independent of the presence of pathology as it did not associate with pathological load. Therefore, a neuroprotective role, rather than a role in promoting disease progression, of Iba1-positive microglia could be hypothesized.

Conflicting results on activated CD68-positive or HLA-DR-positive microglia in DLB phenotypes have been reported previously, in which some studies found an increase in the amygdala, hippocampus, transentorhinal cortex and TC compared to controls [26, 27, 29, 30], while others did not report an increase in the hippocampus or neocortex [31, 32]. Besides, detailed transcriptomic analysis of post-mortem brain tissue in DLB failed to show microglial activation [34, 88]. We only observed a higher reactive microglial load in the CA2 in pure DLB cases compared to controls in our cohort. However, it is important to note that only one study distinguished pure DLB from mixed DLB + AD cases, reporting a higher load in mixed DLB than in pure DLB cases [29]. We found a similar pattern in our cohort, where mixed DLB + AD cases had a higher reactive microglial load than pure DLB cases in the amygdala and PHG. In addition, we confirmed the higher amoeboid and reactive microglial density in mixed DLB when compared to pure DLB cases. The contradicting results on microglial activity in DLB phenotypes are likely related to the fact that pure and mixed DLB cases were not separated in most cohorts. Moreover, results on microglial upregulation in DLB were based on studies with a small sample size, including only 5 DLB cases in each cohort [27, 29, 30], and different techniques for quantification were used between studies. Additionally, we found strong associations between microglial activation and AD-pathology in mixed DLB + AD, pure AD and in pure DLB cases, whereas an association between microglial activation and α-syn pathology did not exist in pure DLB cases. In addition, overall microglial activation was the highest in regions with the highest burden of concomitant α-syn and p-tau pathology in our cohort, i.e. the CA2 and amygdala.

We confirmed significant upregulation of astrocytes in pure AD cases [89, 90]. Two studies in DLB reported an increased astrocytic response in the TC [33] and pulvinar [34] compared to controls, which we could not confirm in our results. However, α-syn accumulation in activated astrocytes has been reported in several studies [11, 37, 91, 92], suggesting a direct link between α-syn accumulation and activation of the innate immune system. Minor astrocytic differences between mixed and pure DLB cases in our cohort suggest astrocytic response to be mostly related to α-syn and not to AD co-pathology, and a region-specific association with α-syn in the amygdala suggests regional vulnerability [11]. Moreover, we did not find a high astroglial load in regions with a high burden of concomitant pathology. It is important to understand that despite being one of the most extensively used astrocytic markers, GFAP only labels the intermediate filament of the cytoskeleton of mature astrocytes, and is therefore not able to stain all astrocytes [93].

Confirming previous studies [94], we found a higher Aβ load in *APOEε4* carriers. In addition, microglial upregulation has previously been described in *APOEε4* carriers [57], which we confirmed in homozygous *APOEε4* carriers. In line with previous results [56], we demonstrated that pathogenic *GBA1* mutation carriers have less AD pathology and are most common in pure phenotypes of DLB. Subtypes of HLA-DRB1*04 were previously described to protect against both PD and AD by improving immune clearance of NFTs [52]. However, we had a total of 11 cases with HLA-DRB1*04 alleles in our cohort only, and were not able to reproduce these findings.

Overall, our study highlights the important role of microglial activation, specifically in mixed cases. Importantly, diverse clinical trials that either influence microglial activation in early disease stages, suppress pro-inflammatory responses of microglia or modulate microglial phenotypic changes to support anti-inflammatory capacities in AD are currently being investigated [95]. In addition, reliable biomarkers to measure AD pathology in CSF exist [96], supporting the opportunity to make an appropriate selection of mixed DLB cases for future clinical trials on immunomodulatory approaches.

Strengths of this study include being one of the first to investigate co-pathology and inflammation in a large number of brain regions in both mixed and pure DLB in a qualitative, semi-quantitative and quantitative manner. Furthermore, multiple neuroinflammatory markers were evaluated, microglia response near plaques using confocal microscopy were studied, and the effects of common genotypes on neuropathology and inflammation were analyzed. However, although we studied a very well-defined cohort, the sample size and clinical information on disease severity were limited. Further, other pathologies, such as TDP-43, and molecular phenotypes of glial cells, to better understand their activation state, were not studied. Future research should focus on spatial transcriptomics to study the molecular phenotype of reactive microglia and astroglia surrounding (co-)pathology. Besides, the quantitative QuPath analyses detected the total %area of immunopositivity, and therefore detected all positive structures. An Artificial Intelligence based approach that would be able to distinguish the different morphological pathological and glial structures would be of great interest for future studies. Finally, studying the level of AD pathology and neuroinflammation via in-vivo biomarkers, e.g., in CSF or plasma, would enhance the investigation of a more comprehensive spectrum of disease stages.

## Conclusion

In conclusion, we highlight that microglial activation in DLB is largely associated with AD co-pathology, whereas astrocytic response did not differ between DLB phenotypes. Most importantly, microglial activity was high in limbic regions, with prevalent AD pathology. We provide novel insights into the molecular neuropathology of DLB and the importance of microglial load, possibly underlying the more rapid disease progression in mixed DLB + AD. Glial biomarkers should be studied to provide insights into disease progression and, more importantly, support the selection of DLB phenotypes for future clinical trials on immunomodulatory approaches.

## Electronic supplementary material

Below is the link to the electronic supplementary material.

Supplementary Material 1

## Data Availability

Supporting data include supplementary tables, supplementary figures, and supplementary material. Accessibility to raw outcome measures will be made available on reasonable request.

## Abbreviations

α-syn: alpha-synuclein
Aβ: amyloid-beta
AD: Alzheimer disease
Amy: amygdala
CA: cornu ammonis
CD68: cluster of differentiation 68
CSF: cerebrospinal fluid
DG: dentate gyrus
DLB: dementia with Lewy bodies
EntC: entorhinal cortex
FusG: fusiform gyrus
GFAP: glial fibrillary acidic protein
GTM: medial temporal gyrus
HipMid: medial hippocampus
HLA-DR: human leukocyte antigen – DR isotype
Iba1: ionized calcium-binding adapter molecule 1
LBs: Lewy bodies
LNs: Lewy neurites
ParaSub: parasubiculum
PD: Parkinson’s disease
PHG: parahippocampal gyrus
p-syn: phosphorylated alpha-synuclein
p-tau: phosphorylated tau
RBD: REM sleep behavior disorders
REM: rapid eye movement
ROI: region of interest
Sub: subiculum
TC: temporal cortex
