# Correction: Neuroinflammation is associated with Alzheimer’s disease co-pathology in dementia with Lewy bodies

**DOI:** 10.1186/s40478-025-02169-8

**Published:** 2025-12-09

**Authors:** Janna van Wetering, Hanne Geut, John J. Bol, Yvon Galis, Evelien Timmermans, Jos W. R. Twisk, Dagmar H. Hepp, Martino L. Morella, Lasse Pihlstrom, Afina W. Lemstra, Annemieke J. M. Rozemuller, Laura E. Jonkman, Wilma D. J. van de Berg

**Affiliations:** 1 Department of Anatomy and Neurosciences, Section Clinical Neuroanatomy and Biobanking and Life Sciences O|2 building 13e55, Amsterdam UMC Location Vrije Universiteit Amsterdam, De Boelelaan 1118, Amsterdam, 1081 HV The Netherlands; 2 Neurodegeneration, Amsterdam Neuroscience, Amsterdam, The Netherlands; 3 Department of Epidemiology and Biostatistics, Amsterdam UMC Location Vrije Universiteit Amsterdam, De Boelelaan 1117, Amsterdam, The Netherlands; 4 Department of Neurology, Leiden University Medical Center, Albinusdreef 2, Leiden, 2333 ZA The Netherlands; 5 Department of Neurology, Oslo University Hospital, Oslo, Norway; 6 Department of Neurology, Amsterdam UMC Location Vrije Universiteit Amsterdam, De Boelelaan 1117, Amsterdam, The Netherlands; 7 Alzheimer Center, Department of Neurology, Amsterdam UMC, Vrije Universiteit Amsterdam, Amsterdam Neuroscience, Amsterdam, The Netherlands; 8 Department of Pathology, Amsterdam UMC Location Vrije Universiteit Amsterdam, De Boelelaan 1117, Amsterdam, The Netherlands

**Correction to: Acta Neuropathologica Communications (2024) 12:73** 10.1186/s40478-024-01786-z

In this article [1], the given and family names of Janna van Wetering were incorrectly structured. The name was displayed correctly in all versions at the time of publication.

The original article has been corrected.
