# Supplementary material for: Neuroinflammation is associated with Alzheimer’s disease co-pathology in dementia with Lewy bodies
Source: Acta Neuropathol Commun. 2024 May 7;12:73. doi: 10.1186/s40478-024-01786-z (PMC11075309; doi:10.1186/s40478-024-01786-z)
Supplement: Supplementary file 1 — Supplementary Material 1 [file 40478_2024_1786_MOESM1_ESM.docx]

|  | Supplement 1. Case summaries. N.a. = not available | | | | | | | | | | | | | | | |
| --- | --- | --- | --- | --- | --- | --- | --- | --- | --- | --- | --- | --- | --- | --- | --- | --- |
| Donor | | **Neuro-pathological diagnosis** | **Sex** | **Age at onset** | **Age at death** | **Cause of death** | **PMD (hrs:min)** | **Braak α-syn stage [1]** | **Thal amyloid-beta stage [2]** | **Braak NFT stage [3]** | **CERAD [4]** | **CAA type [5]** | ***APOE*** | ***HLA-DRB1*04***  **alleles** | ***GBA1* mutation** |  |
| 1 | | Control | M |  | 68 | euthanasia | 8:40 | 0 | 2 | 1 | none | 2 | 3 / 4 | 1 | n.a. |  |
| 2 | | Control | F |  | 63 | euthanasia | 8:00 | 0 | 0 | 0 | none | 0 | 2 / 3 | 1 | n.a. |  |
| 3 | | Control | F |  | 72 | decompensatio cordis | 8:35 | 0 | 0 | 0 | none | 0 | 3 / 3 | 0 | n.a. |  |
| 4 | | Control | F |  | 69 | pulmonary embolism | 7:20 | 0 | 1 | 1 | none | 0 | 3 / 3 | 0 | n.a. |  |
| 5 | | Control | M |  | 59 | euthanasia | 13:00 | 0 | 2 | 1 | none | 0 | 3 / 4 | n.a. | n.a. |  |
| 6 | | Control | M |  | 77 | pneumonia | 11:45 | 0 | 1 | 1 | none | 0 | 2 / 3 | 1 | n.a. |  |
| 7 | | Control | F |  | 79 | unknown | 5:25 | n.a. | 3 | 2 | none |  | 3 / 3 | 0 | n.a. |  |
| 8 | | Control | F |  | 78 | unknown | 10:00 | 0 | 1 | 1 | none | 0 | 3 / 3 | 0 | n.a. |  |
| 9 | | Control | F |  | 59 | euthanasia | 4:30 | 0 | 0 | 0 | none | 0 | 3 / 3 | 0 | n.a. |  |
| 10 | | Control | F |  | 71 | lung carcinoma | 8:10 | 0 | 2 | 1 | none | 1 | 3 / 4 | 0 | n.a. |  |
| 11 | | Control | M |  | 74 | euthanasia | 10:20 | 0 | 3 | 2 | none | 0 | 3 / 3 | 2 | n.a. |  |
| 12 | | pure AD | F | 84 | 92 | decompensatio cordis | 7:00 | 0 | 4 | 5 | frequent | 1 | 3 / 4 | n.a. | n.a. |  |
| 13 | | pure AD | F | 75 | 84 | cachexia and cardiac arrest | 6:30 | 0 | 4 | 5 | frequent | 1 | 3 / 4 | n.a. | n.a. |  |
| 14 | | pure AD | F | 74 | 89 | cardiogenic shock | 4:40 | 0 | 4 | 5 | frequent | 1 | 3 / 4 | n.a. | n.a. |  |
| 15 | | pure AD | M | 63 | 70 | metastasized colon carcinoma | 6:20 | 0 | 4 | 6 | frequent | 1 | 3 / 4 | n.a. | n.a. |  |
| 16 | | pure AD | M | 77 | 88 | cachexia and dehydration | 5:30 | 0 | 4 | 6 | moderate | 1 | 3 / 4 | n.a. | n.a. |  |
| 17 | | pure AD | M | 58 | 74 | cachexia and dehydration | 3:05 | 0 | 5 | 6 | frequent | 2 | 4 / 4 | n.a. | n.a. |  |
| 18 | | pure AD | M | 58 | 60 | euthanasia | 8:21 | 0 | 5 | 6 | frequent | 1 | 3 / 3 | n.a. | n.a. |  |
| 19 | | pure AD | M | 62 | 68 | euthanasia | 9:09 | 0 | 5 | 5 | frequent | 2 | 3 / 3 | n.a. | n.a. |  |
| 20 | | pure AD | F | 58 | 69 | pulmonary infection | 11:33 | 0 | 5 | 5 | frequent | 1 | 3 / 4 | n.a. | n.a. |  |
| 21 | | pure AD | F | 73 | 80 | euthanasia | 7:03 | 0 | 5 | 4 | moderate | 1 | 3 / 3 | n.a. | n.a. |  |
| 22 | | pure AD | M | 48 | 53 | palliative sedation | 9:00 | 0 | 5 | 6 | frequent | 1 | 3 / 3 | n.a. | n.a. |  |
| 23 | | pure AD | M | 52 | 64 | cachexia and dehydration | 5:17 | 0 | 5 | 6 | frequent | 2 | 3 / 4 | n.a. | n.a. |  |
| 24 | | pure AD | M | 61 | 84 | euthanasia | 6:16 | 0 | 3 | 4 | moderate |  | 3 / 3 | n.a. | n.a. |  |
| 25 | | pure AD | M | 67 | 77 | suicide by drugs | 6:17 | 0 | 5 | 6 | frequent | 1 | 4 / 4 | n.a. | n.a. |  |
| 26 | | pure AD | M | 58 | 65 | euthanasia | 9:18 | 0 | 5 | 5 | frequent | 1 | 3 / 4 | n.a. | n.a. |  |
| 27 | | pure AD | M | 71 | 84 | unknown | 5:53 | 0 | 5 | 4 | moderate | 1 | 3 / 4 | n.a. | n.a. |  |
| 28 | | pure DLB | M | 63 | 70 | cachexia | 4:25 | 6 | 2 | 2 | sparse | 2 | 3 / 4 | 1 | no |  |
| 29 | | pure DLB | F | 53 | 61 | abdominal problems | 4:30 | 6 | 3 | 1 | none | 0 | 3 / 4 | 0 | E326K / |  |
| 30 | | pure DLB | M | 60 | 66 | unknown | 4:30 | 5 | 0 | 1 | none | 0 | n.a. | n.a. | n.a. |  |
| 31 | | pure DLB | M | 68 | 73 | unknown | 5:35 | 6 | 3 | 1 | sparse | 1 | n.a. | n.a. | n.a. |  |
| 32 | | pure DLB | M | 71 | 78 | cachexia and dehydration | 4:30 | 6 | 0 | 2 | none | 2 | 3 / 3 | 0 | E326K / |  |
| 33 | | pure DLB | M | 60 | 72 | cachexia | 4:30 | 6 | 0 | 2 | none | 0 | 2 / 3 | 1 | E326K / |  |
| 34 | | pure DLB | M | 59 | 67 | pneumonia | 6:30 | 6 | 0 | 2 | none | 0 | 3 / 3 | 0 | N370S / |  |
| 35 | | pure DLB | M | 75 | 83 | urinary tract infection | 4:45 | 6 | 2 | 1 | sparse | 0 | 3 / 4 | 0 | no |  |
| 36 | | pure DLB | F | 74 | 81 | cachexia | 6:30 | 5 | 2 | 2 | none | 1 | 3 / 3 | 0 | T369M / |  |
| 37 | | pure DLB | M | 66 | 71 | stroke | 5:05 | 6 | 4 | 2 | moderate | 1 | 3 / 3 | 0 |  |  |
| 38 | | pure DLB | M | 76 | 79 | cachexia and dehydration | 4:00 | 6 | 2 | 1 | sparse | 1 | 3 / 4 | 1 | n.a. |  |
| 39 | | pure DLB | M | 70 | 83 | pneumonia | 4:30 | 6 | 0 | 1 | none | 0 | 3 / 3 | 1 | no |  |
| 40 | | pure DLB | M | 79 | 90 | decompensatio cordis | 4:05 | 6 | 3 | 1 | sparse | 2 | 3 / 3 | 0 | N370S / |  |
| 41 | | pure DLB | M | 73 | 84 | pneumonia | 4:50 | 6 | 3 | 2 | none | 2 | 3 / 4 | 0 | T369M / |  |
| 42 | | pure DLB | F | 62 | 74 | pneumonia | 6:35 | 6 | 1 | 2 | none | 0 | 3 / 3 | 0 | T369M / |  |
| 43 | | mixed DLB+AD | M | 76 | 79 | unknown | 2:15 | 6 | 3 | 4 | moderate | 2 | n.a. | n.a. | n.a. |  |
| 44 | | mixed DLB+AD | M | 63 | 71 | unknown | 3:00 | 6 | 3 | 4 | sparse | 2 | n.a. | n.a. | n.a. |  |
| 45 | | mixed DLB+AD | F | 77 | 82 | unknown | 3:55 | 6 | 4 | 4 | sparse | 1 | 3 / 4 | 0 | no |  |
| 46 | | mixed DLB+AD | M | 77 | 81 | stomach bleeding | 5:45 | 6 | 3 | 4 | sparse | 2 | 3 / 4 | n.a. | no |  |
| 47 | | mixed DLB+AD | M | 81 | 86 | pneumonia | 7:52 | 5 | 3 | 5 | sparse | 3 | 3 / 4 | n.a. | no |  |
| 48 | | mixed DLB+AD | M | 72 | 74 | unknown sudden death | 5:25 | 5 | 4 | 4 | moderate | 1 | 3 / 4 | 0 | no |  |
| 49 | | mixed DLB+AD | F | 74 | 82 | cachexia and dehydration | 4:05 | 6 | 4 | 6 | moderate | 1 | 2 / 4 | 0 | no |  |
| 50 | | mixed DLB+AD | F | 59 | 70 | cachexia and dehydration | 4:20 | 5 | 4 | 6 | frequent | 2 | 3 / 4 | 0 | no |  |
| 51 | | mixed DLB+AD | F | 66 | 71 | dehydration | 4:35 | 5 | 3 | 4 | sparse | 1 | 2 / 4 | 0 | no |  |
| 52 | | mixed DLB+AD | M | 68 | 73 | pneumonia | 4:10 | 6 | 4 | 5 | sparse | 1 | 3 / 4 | 0 | no |  |
| 53 | | mixed DLB+AD | M | 64 | 73 | sepsis | 4:55 | 6 | 4 | 5 | moderate | 2 | 3 / 4 | n.a. | no |  |
| 54 | | mixed DLB+AD | M | 80 | 86 | cachexia and dehydration | 6:45 | 6 | 4 | 3 | sparse | 1 | 3 / 4 | n.a. | no |  |
| 55 | | mixed DLB+AD | M | 67 | 73 | urosepsis | 6:30 | 6 | 3 | 4 | sparse | 1 | 3 / 4 | 0 | E326K / |  |
| 56 | | mixed DLB+AD | F | 79 | 87 | cachexia | 5:15 | 6 | 4 | 4 | sparse | 1 | 3 / 4 | 0 | no |  |
| 57 | | mixed DLB+AD | M | 70 | 75 | dehydration | 4:25 | 6 | 3 | 3 | moderate | 2 | 3 / 4 | 0 | no |  |
| 58 | | mixed DLB+AD | M | 67 | 72 | aspiration pneumonia | 6:40 | 6 | 3 | 4 | moderate | 2 | 3 / 4 | 0 | no |  |
| 59 | | mixed DLB+AD | M | 60 | 62 | cachexia and dehydration | 4:35 | 6 | 4 | 4 | sparse | 1 | 3 / 4 | 0 | V460M / |  |
| 60 | | mixed DLB+AD | M | 76 | 81 | pneumonia and sepsis | 4:30 | 5 | 4 | 4 | frequent | 3 | 3 / 4 | 1 | T369M / |  |
| 61 | | mixed DLB+AD | F | 79 | 83 | infection bowel or urinary tract | 6:15 | 6 | 3 | 4 | sparse | 1 | 3 / 3 | 0 |  |  |
| 62 | | mixed DLB+AD | M | 76 | 79 | dehydration | 4:25 | 6 | 3 | 4 | moderate | 2 | 3 / 4 | n.a. | no |  |
| 63 | | mixed DLB+AD | F | 70 | 81 | cachexia and dehydration | 5:15 | 6 | 4 | 4 | sparse | 1 | 3 / 4 | 2 | no |  |
| 64 | | mixed DLB+AD | F | 72 | 82 | pneumonia | 5:10 | 6 | 3 | 5 | moderate | 1 | 3 / 4 | 0 | no |  |
| 65 | | mixed DLB+AD | F | 67 | 71 | pneumonia | 5:40 | 6 | 4 | 5 | moderate | 1 | 4 / 4 | 0 | no |  |
| 66 | | mixed DLB+AD | M | 65 | 69 | pneumonia | 4:40 | 6 | 4 | 4 | sparse | 1 | 3 / 4 | 0 | E326K / |  |
| 67 | | mixed DLB+AD | M | 61 | 69 | cachexia | 4:45 | 6 | 3 | 4 | frequent | 2 | 3 / 4 | 0 | no |  |
| 68 | | mixed DLB+AD | M | 60 | 74 | aspiration pneumonia | 4:25 | 5 | 2 | 3 | moderate | 1 | 3 / 4 | 0 | no |  |
| 69 | | mixed DLB+AD | M | 72 | 79 | aspiration pneumonia and cachexia | 5:48 | 6 | 4 | 4 | sparse | 1 | 3 / 4 | 0 | no |  |
| 70 | | mixed DLB+AD | F | 75 | 81 | cachexia and dehydration | 3:35 | 6 | 4 | 5 | moderate | 2 | 3 / 3 | 0 | no |  |
| 71 | | mixed DLB+AD | M | 50 | 62 | urinary tract infection | 6:05 | 6 | 4 | 4 | moderate | 1 | 3 / 4 | 0 | no |  |
| 72 | | mixed DLB+AD | M | 63 | 71 | voluntarily stopping eating | 6:00 | 6 | 4 | 4 | frequent | 1 | 3 / 4 | 0 | no |  |
| 73 | | mixed DLB+AD | F | 62 | 68 | delirium | 4:35 | 6 | 4 | 4 | sparse | 1 | 4 / 4 | 0 | no |  |
| 74 | | mixed DLB+AD | M | 60 | 63 | hepatic insufficiency | 4:55 | 6 | 4 | 4 | frequent | 1 | 4 / 4 | 1 | no |  |
| 75 | | mixed DLB+AD | M | 62 | 67 | pneumonia | 5:20 | 6 | 4 | 5 | frequent | 1 | 4 / 4 | 0 | no |  |
| 76 | | mixed DLB+AD | M | 51 | 56 | cachexia and dehydration | 5:00 | 6 | 4 | 5 | frequent | 2 | 3 / 4 | 0 | no |  |
| 77 | | mixed DLB+AD | F | 79 | 84 | cachexia and delirium | 4:10 | 6 | 4 | 5 | frequent | 2 | 3 / 3 | 0 | no |  |

**Supplement 2.** Staining protocols

*Immunohistochemistry staining protocols*

For alpha-synuclein (KM51) and amyloid-beta (6F/3D) staining, after deparaffinization and rinsing in citrate buffer pH 6.0, antigen retrieval was performed for 10 minutes in a citrate buffer (pH 6.0) in a microwave oven. Consequently, the slides were rinsed in distilled water, and a second antigen retrieval step was performed with 80% formic acid and PBS. The sections were incubated in alpha-synuclein or amyloid-beta overnight at 4ºC. Afterwards, the slides were incubated with the secondary antibody from the Envision-kit for 1 hour and DAB chromogen was consecutively applied to the slides. Ultimately, a haematoxylin staining was performed, sections were mounted using Entellan (*Sigma-Aldrich*) and a coverslip was applied to all sections.

The phosphorylated-tau (AT8) staining was performed similarly. However, after deparaffinization, sections were rinsed in TBS and the antigen step was performed in TBS (pH 9.0). Consequently, slides were rinsed in PBS and incubated in the p-tau for 2 hours at room temperature or overnight at 4ºC. Subsequently, the protocol was identical to the KM51 and 6F/3D protocol.

For the CD68, IBA1 and GFAP staining, after pre-heating treatment and deparaffinization, antigen retrieval was performed for 30 minutes in Tris-EDTA buffer (pH 9.0) in a steamcooker. Subsequently, for the CD68 and the IBA1 staining the slides were incubated in 1% hydrogen peroxide in TBS to block endogenous peroxidase and 5% non-fat dry milk in TBS with 0.1% Triton X-100 (TBS-T) to block unspecific staining. For the GFAP staining 5% non-fat dry milk was substituted for 3% normal donkey serum. The sections were incubated in CD68, IBA1 or GFAP overnight at 4 °C. Afterwards, the slides were incubated with the secondary antibodies and the ABC-kit *(1/400, Catalogue No. PK6100, Vector)* in TBS-T and DAB chromogen was consecutively applied to the slides. Finally, a staining with haematoxylin was performed to visualize all nuclei, after which sections were dehydrated and coverslipped with Entellan (*Sigma-Aldrich*).

The HLA-DR staining was performed similarly. However, TBS was replaced by PBS, the antigen retrieval step was performed in autoclave and the used buffer was Citrate (pH 6.0). The secondary antibody used was Envision.

*Multi-labeling immunofluorescence staining protocols*

For the multi-labeling protocol, after pre-heating and deparaffinization, antigen retrieval was performed for 30 minutes in citrate buffer (pH 6.0) in a steamcooker and for 5 minutes in 80% formic acid. Subsequently, the slides were incubated in 1% hydrogen peroxide in TBS to block endogenous peroxidase and 3% Normal Donkey Serum in TBS + 0.1% Triton x-100 (BB) to block unspecific staining. The sections were incubated in amyloid-beta overnight at 4 °C. After incubation with mouse-Envision for 30 minutes, the sections were stained with Tyramid Alexa 555 for 20 minutes. Afterwards, antibody removal step was performed in a steamcooker for 10 minutes in citrate buffer (pH 6.0) and the slides were blocked in BB and incubated in the primary antibodies (IBA1 + p-tau + p-syn) overnight at 4 °C. Primary antibodies were detected and visualized with Alexa 647, Alexa 594 and Alexa 488 respectively. After counterstaining with DAPI (*Sigma-Aldrich, Missouri, United States)*, sections were mounted with Mowiol plus DABCO.

| **Summary of antibodies used in immunohistochemistry and multi-labeling experiments** | | | | | |
| --- | --- | --- | --- | --- | --- |
| **Immunohistochemistry (IHC)** | | | | | |
| **Primary antibody** | **Clone** | **Antibody ID** | **Source** | **Dilution** | **Host** |
| Alpha-synuclein | KM51 | NCL-L-ASYN; MONX10739 | Novocastra, Leica AND Monosan Xtra, The Netherlands | 1/500 | Mouse |
| Amyloid-beta | 6F/3D | M087201 | DAKO, Denmark | 1/500 | Mouse |
| Phosphorylated-tau | AT8 | MN1020 | Thermo Fisher Scientific, USA | 1/500 | Mouse |
| Iba1 | IBA1 | AB5076 | Abcam | 1/2000 | Goat |
| CD68 | KP1 | M0814 | DAKO, Denmark | 1/500 | Mouse |
| HLA-DR | CR3/43 | M0775 | DAKO, Denmark | 1/400 | Mouse |
| GFAP | GFAP | G3893 | SIGMA | 1/1000 | Mouse |
| **Secondary antibody** | **Clone** | **Antibody ID** | **Source** | **Dilution** | **Host** |
| Biotinylated donkey anti-mouse IgG |  | 715-065-151 | Jackson | 1/400 |  |
| Biotinylated donkey anti-goat IgG |  | 705-065-147 | Jackson | 1/400 |  |
| **Immunofluorescence (multi-labeling)** | | | | | |
| **Primary antibody** | **Clone** | **Antibody ID** | **Source** | **Dilution** | **Host** |
| Alpha-synuclein | EP1536Y | AB51253 | Abcam | 1/500 | Rabbit |
| Amyloid-beta | 6F/3D | M087201 | DAKO, Denmark | 1/1000 | Mouse |
| Phosphorylated-tau | AT8 | MN1020 | Thermo Fisher Scientific, USA | 1/400 | Mouse |
| Iba1 | IBA1 | AB5076 | Abcam | 1/200 | Goat |
| **Secondary antibody** | **Fluorescence** | **Antibody ID** | **Source** | **Dilution** | **Host** |
| Iba1; donkey anti-goat | Alexa 647 | *A32849* | *Mol. Probes* | 1/200 |  |
| Phosphorylated-tau; donkey anti-mouse | Alexa 594 | *A21203* | *Mol. Probes* | 1/200 |  |
| Alpha-synuclein; donkey anti-rabbit | Alexa 488 | *A32790* | *Invitrogen* | 1/400 |  |
| Amyloid-beta; anti-mouse | Alexa 555 tyramide | *B40955* | *Invitrogen* | 1/100 |  |

*Confocal microscopy*

Confocal imaging was performed on a Leica TCS SP8 (Leica, Microsystems, Germany) using a HCL PL PAO CS2 63x oil objective lens, NA 1.40 and a zoom factor depending on the size of the structure of interest. A pulsed white light laser at different wavelengths (excitation wavelenghts: DAPI at 405 nm; Alexa 555 at 547 nm; Alexa 647 at 653 nm; Alexa 594 at 590 nm; Alexa 488 at 499 nm) was used to sequentially scan sections for each fluorochrome. Signals were detected using gated hybrid detectors in counting mode. Z-stacks were taken in the CA1 and EntC of representative mixed DLB+AD cases. Afterwards, images were deconvoluted using standard CLSM algorithms in Huygens Professional (Scientific Volume imaging; Huygens, The Netherlands; <https://svi.nl/Huygens-Professional>), and their maximum projections (ImageJ Fiji, National Institute of Health USA; <https://imagej.nih.gov/ij/>) were used to represent graphically the structures of interests and their co-localization. ImageJ was used in some cases to adjust brightness and figures were created using Adobe Illustrator (CS6, Adobe Systems incorporated).


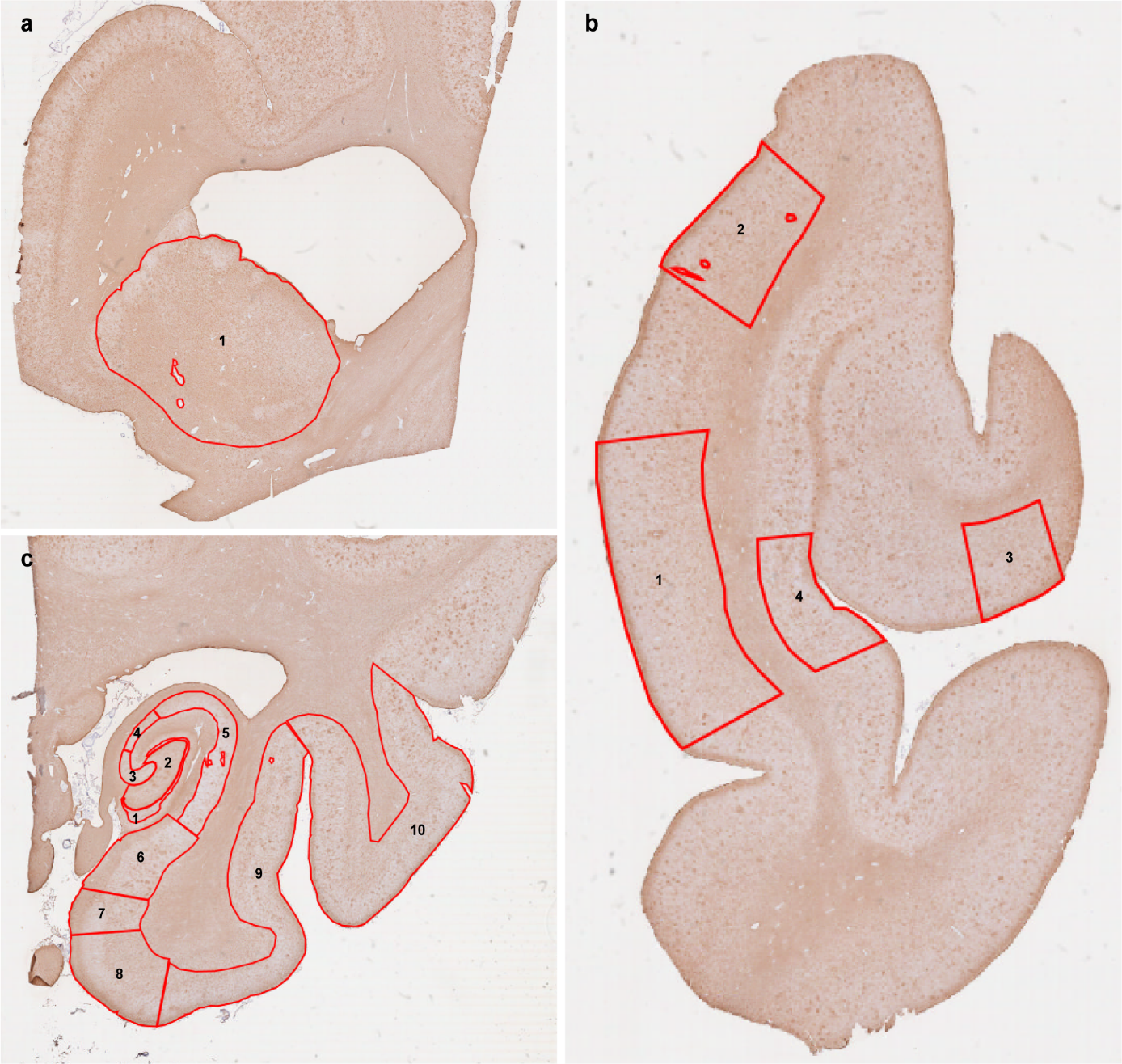


**Supplement 3.** Examples of region of interest (ROI) delineation of the amygdala, hippocampus and cortical regions. **a.1** Delineation of the amygdaloid complex. **b.1-4** Delineation of different ROIs in the temporal cortex. For immunopositivity, mean outcome measurement of different ROIs was calculated. **c1-7** Delineation of the hippocampus. **c.1** Dentate gyrus (DG). **c.2** Cornu Ammonis 4 (CA). **c.3** CA3. **c.4** CA2. **c.5** CA1. **c.6** Subiculum (Sub). **c.7** Parasubiculum (ParaSub). **c8-10** Delineation of remaining cortical regions. **c.8** Entorhinal cortex (EntC). **c.9** Parahippocampal gyrus (PHG). **c.10** Fusiform gyrus (FusG).

**Supplement 4.** QuPath scripts for quantification of glial load

All scripts for quantifying glial cells are based on the methodology outlined in the article by Finney et al. [6], which quantified IHC staining of astrocytes based on pixel load, and adjusted for the performed stainings in post-mortem brain tissue. Glial load was defined as percentage area of immunopositive pixels of CD68 and HLA-DR (active microglia), Iba1 (ramified microglia) and GFAP (reactive astrocytes). In short, for microglial and astrocytic markers, the DAB channel was selected by setting Color Deconvolution Stains and background staining was subtracted for all images using the exact values added in the script below. For α-syn-, Aβ- and p-tau-markers, the DAB channel was manually selected as ‘auto’ per image, due to varying background staining between images within a staining. Following, the manually drawn regions of interest were selected. Per script, Gaussian sigma and thresholds were set as manually determined on beforehand by sampling different cases for all stainings.

*Script α-syn load:*

selectAnnotations();
runPlugin('qupath.imagej.detect.tissue.PositivePixelCounterIJ', '{"downsampleFactor": 0.1, "gaussianSigmaMicrons": 0.1, "thresholdStain1": 20.0, "thresholdStain2": 0.4, "addSummaryMeasurements": true, "clearParentMeasurements": true, "appendDetectionParameters": true, "legacyMeasurements0.1.2": false}'); saveAnnotationMeasurements(folder).

*Script Aβ load:*

selectAnnotations();
runPlugin('qupath.imagej.detect.tissue.PositivePixelCounterIJ', '{"downsampleFactor": 0.10, "gaussianSigmaMicrons": 0.5, "thresholdStain1": 20.0, "thresholdStain2": 0.3, "addSummaryMeasurements": true, "clearParentMeasurements": true, "appendDetectionParameters": true, "legacyMeasurements0.1.2": false}'); saveAnnotationMeasurements(folder).

*Script p-tau load:*

selectAnnotations();
runPlugin('qupath.imagej.detect.tissue.PositivePixelCounterIJ', '{"downsampleFactor": 0.1, "gaussianSigmaMicrons": 0.1, "thresholdStain1": 200.0, "thresholdStain2": 0.5, "addSummaryMeasurements": true, "clearParentMeasurements": true, "appendDetectionParameters": true, "legacyMeasurements0.1.2": false}'); saveAnnotationMeasurements(folder).

*Script Iba1 load*:

setImageType('BRIGHTFIELD_H_DAB');
setColorDeconvolutionStains('{"Name" : "H-DAB IBA1", "Stain 1" : "Hematoxylin", "Values 1" : "0.75607 0.6231 0.20025 ", "Stain 2" : "DAB", "Values 2" : "0.30283 0.56722 0.76587 ",
"Background":"240 241 227"}');
selectAnnotations(); runPlugin('qupath.imagej.detect.tissue.PositivePixelCounterIJ', '{"downsampleFactor": 1, "gaussianSigmaMicrons": 0.3, "thresholdStain1": 5000.0, "thresholdStain2": 0.2, "addSummaryMeasurements": true, "clearParentMeasurements": true, "appendDetectionParameters": false, "legacyMeasurements0.1.2": false}'); saveAnnotationMeasurements(folder).

*Script HLA-DR load:*

setImageType('BRIGHTFIELD_H_DAB');
setColorDeconvolutionStains ('{"Name" : "CR343", "Stain 1" : "Hematoxylin", "Values 1" : "0.64574 0.64568 0.40757 ", "Stain 2" : "DAB", "Values 2" : "0.35733 0.57507 0.73594 ",
"Background":"241 236 237"}');
selectAnnotations(); runPlugin('qupath.imagej.detect.tissue.PositivePixelCounterIJ', '{"downsampleFactor": 1, "gaussianSigmaMicrons": 0.1, "thresholdStain1": 5000.0, "thresholdStain2": 0.1, "addSummaryMeasurements": true, "clearParentMeasurements": true, "appendDetectionParameters": true, "legacyMeasurements0.1.2": false}'); saveAnnotationMeasurements(folder).

*Script CD68 load:*

setImageType('BRIGHTFIELD_H_DAB');
setColorDeconvolutionStains('{"Name" : "H-DAB default", "Stain 1" : "Hematoxylin", "Values 1" : "0.65111 0.70119 0.29049 ", "Stain 2" : "DAB", "Values 2" : "0.26917 0.56824 0.77759 ", "Background":"255 255 255"}');
selectAnnotations(); runPlugin('qupath.imagej.detect.tissue.PositivePixelCounterIJ', '{"downsampleFactor": 1, "gaussianSigmaMicrons": 0.1, "thresholdStain1": 5000.0, "thresholdStain2": 0.22, "addSummaryMeasurements": true, "clearParentMeasurements": true, "appendDetectionParameters": false, "legacyMeasurements0.1.2": false}');
saveAnnotationMeasurements(folder).

*Script GFAP load:*

setImageType('BRIGHTFIELD_H_DAB');
setColorDeconvolutionStains('{"Name" : "H-DAB IBA1", "Stain 1" : "Hematoxylin", "Values 1" : "0.75607 0.6231 0.20025 ", "Stain 2" : "DAB", "Values 2" : "0.30283 0.56722 0.76587 ", "Background" :"240 241 227 "}');
selectAnnotations(); runPlugin('qupath.imagej.detect.tissue.PositivePixelCounterIJ', '{"downsampleFactor": 1, "gaussianSigmaMicrons": 0.3, "thresholdStain1": 5000.0, "thresholdStain2": 0.2, "addSummaryMeasurements": true, "clearParentMeasurements": true, "appendDetectionParameters": true, "legacyMeasurements0.1.2": false}'); saveAnnotationMeasurements(folder)

**Supplement 5. Detection of antibody immunopositivity by in-house developed QuPath scripts** **a** Immunostaining against full-length alpha-synuclein (KM51), demonstrating specific neuronal, and dot-like synaptic and astrocytic alpha-synuclein in the neuropil. **c** Immunostaining against amyloid-beta (6F/3D) demonstrated diffuse neuritic and classic-cored amyloid-beta plaques. **e** Immunostaining against phosphorylated-tau (AT8) showed neurofibrillary tangles, neuropil threads, dot-like synaptic tau staining and accumulated phosphorylated tau.  **g** Immunostaining against total microglia (Iba1) demonstrated mainly homeostatic microglia with thin processes and some amoeboid and reactive microglia with a larger soma.  **i** Immunostaining against microglia (HLA-DR) demonstrated microglia with a large soma and short, thick processes. **k** Immunostaining against microglia (CD68) demonstrated amoeboid microglia without processes, making it difficult to interpret the morphology. **m** Immunostaining against astrocytes (GFAP) stained astrocytic cell bodies with many long processes and many dot-like or complex GFAP-positive processes from which the cell body could not be detected within the 6 um section of brain tissue. **b,d,f,h,j,l,n** Shown in red, specific immunopositive staining identified as ‘positive’ by the respective QuPath script, based on pixel quantification.


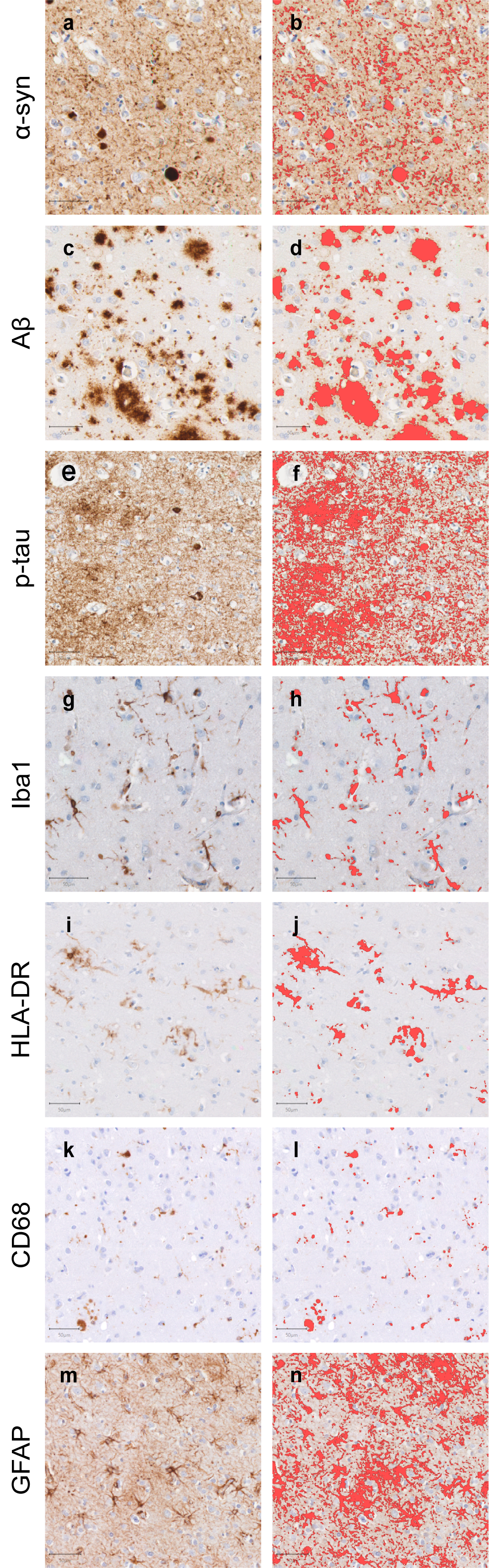


**Supplement 6.** Estimates of group differences in α-syn, Aβ and p-tau pathology that resulted from study design were not named in results section of the manuscript, and are therefore stated as following. By design, mixed DLB+AD cases had a higher α-syn load when compared to pure AD cases in CA2, PHG, amygdala (all *p* < 0.001) and CA3 (*p* = 0.036). Aβ load was higher in mixed DLB+AD than in pure DLB cases in the parasubiculum, EntC, PHG, FusG and TC (*p* < 0.001). Besides, p-tau load was significantly higher in mixed DLB+AD cases than in pure DLB in the CA1, CA2, subiculum, EntC, PHG, FusG (*p* < 0.001), CA3, TC and amygdala (*p* = 0.011, *p* = 0.014, *p* = 0.004).

**Supplement 7.** Estimates of regional differences in α-syn, Aβ and p-tau pathology, micro- and astroglial load. Brain region in vertical column on the left is compared with each brain region displayed horizontally at the top of the table. Analyses were made in a mixed-effects linear regression. Coefficient and p-value are reported within the table. P-values that remained < 0.05 after FDR-correction for multiple corrections were considered significant and are reported in **bold.**

| **Alpha-synuclein load** | | **EntC** | **PHG** | **FusG** | **TC** | **Amy** | **CA1** | **CA2** | **CA3** | **CA4** | **DG** | **Sub** | **Para- Sub** |
| --- | --- | --- | --- | --- | --- | --- | --- | --- | --- | --- | --- | --- | --- |
| **EntC** | Coefficient |  | **1.107** | 0.598 | 0.484 | **1.053** | 0.385 | **3.615** | **0.806** | 0.027 | -0.199 | -0.103 | -0.118 |
|  | p-value |  | **0.000** | 0.064 | 0.116 | **0.001** | 0.214 | **0.000** | **0.010** | 0.931 | 0.521 | 0.740 | 0.704 |
| **PHG** | Coefficient | **-1,107** |  | -0.509 | -0.623 | -0.054 | **-0.722** | **2.508** | -0.301 | **-1.080** | **-1.306** | **-1.210** | **-1.225** |
|  | p-value | **0,000** |  | 0.119 | 0.047 | 0.867 | **0.022** | **0.000** | 0.343 | **0.001** | **0.000** | **0.000** | **0.000** |
| **FusG** | Coefficient | -0,598 | 0.509 |  | -0.113 | 0.456 | -0.212 | **3.018** | 0.208 | -0.571 | **-0.797** | -0.701 | -0.716 |
|  | p-value | 0,064 | 0.119 |  | 0.725 | 0.165 | 0.512 | **0.000** | 0.523 | 0.080 | **0.014** | 0.030 | 0.028 |
| **TC** | Coefficient | -0,484 | 0.623 | 0.113 |  | 0.569 | -0.099 | **3.131** | 0.322 | -0.457 | -0.684 | -0.587 | -0.603 |
|  | p-value | 0,116 | 0.047 | 0.725 |  | 0.069 | 0.749 | **0.000** | 0.302 | 0.143 | 0.027 | 0.058 | 0.052 |
| **Amy** | Coefficient | **-1,053** | 0.054 | -0.456 | -0.569 |  | -0.668 | **2.562** | -0.247 | **-1.026** | **-1.253** | **-1.157** | **-1.172** |
|  | p-value | **0,001** | 0.867 | 0.165 | 0.069 |  | 0.035 | **0.000** | 0.438 | **0.001** | **0.000** | **0.000** | **0.000** |
| **CA1** | Coefficient | -0,385 | 0.722 | 0.212 | 0.099 | 0.668 |  | **3.230** | 0.421 | -0.358 | -0.585 | -0.489 | -0.504 |
|  | p-value | 0,214 | 0.022 | 0.512 | 0.749 | 0.035 |  | **0.000** | 0.180 | 0.253 | 0.060 | 0.116 | 0.107 |
| **CA2** | Coefficient | **-3,615** | **-2.508** | **-3.018** | **-3.131** | **-2.562** | **-3.230** |  | **-2.809** | **-3.588** | **-3.815** | **-3.719** | **-3.734** |
|  | p-value | **0,000** | **0.000** | **0.000** | **0.000** | **0.000** | **0.000** |  | **0.000** | **0.000** | **0.000** | **0.000** | **0.000** |
| **CA3** | Coefficient | **-0,806** | 0.301 | -0.208 | -0.322 | 0.247 | -0.421 | **2.809** |  | **-0.779** | **-1.005** | **-0.909** | **-0.924** |
|  | p-value | **0,010** | 0.343 | 0.523 | 0.302 | 0.438 | 0.180 | **0.000** |  | **0.014** | **0.001** | **0.004** | **0.003** |
| **CA4** | Coefficient | -0,027 | **1.080** | 0.571 | 0.457 | **1.026** | 0.358 | **3.588** | **0.779** |  | -0.226 | -0.130 | -0.145 |
|  | p-value | 0,931 | **0.001** | 0.080 | 0.143 | **0.001** | 0.253 | **0.000** | **0.014** |  | 0.470 | 0.678 | 0.645 |
| **DG** | Coefficient | 0,199 | **1.306** | **0.797** | **0.684** | **1.253** | 0.585 | **3.815** | **1.005** | 0.226 |  | 0.096 | 0.081 |
|  | p-value | 0,521 | **0.000** | **0.014** | **0.027** | **0.000** | 0.060 | **0.000** | **0.001** | 0.470 |  | 0.758 | 0.796 |
| **Sub** | Coefficient | 0,103 | **1.210** | 0.701 | 0.587 | **1.157** | 0.489 | **3.719** | **0.909** | 0.130 | -0.096 |  | -0.015 |
|  | p-value | 0,740 | **0.000** | 0.030 | 0.058 | **0.000** | 0.116 | **0.000** | **0.004** | 0.678 | 0.758 |  | 0.961 |
| **ParaSub** | Coefficient | 0,118 | **1.225** | 0.716 | 0.603 | **1.172** | 0.504 | **3.734** | **0.924** | 0.145 | -0.081 | 0.015 |  |
|  | p-value | 0,704 | **0.000** | 0.028 | 0.052 | **0.000** | 0.107 | **0.000** | **0.003** | 0.645 | 0.796 | 0.961 |  |
| **Amyloid-beta load** | | **EntC** | **PHG** | **FusG** | **TC** | **Amy** | **CA1** | **CA2** | **CA3** | **CA4** | **DG** | **Sub** | **Para- Sub** |
| **EntC** | Coefficient |  | 0.075 | 0.386 | 0.304 | **-1.676** | **-2.117** | **-2.657** | **-2.808** | **-2.796** | **-2.444** | **-1.846** | 0.101 |
|  | p-value |  | 0.802 | 0.212 | 0.320 | **0.000** | **0.000** | **0.000** | **0.000** | **0.000** | **0.000** | **0.000** | 0.741 |
| **PHG** | Coefficient | -0,075 |  | 0.311 | 0.229 | **-1.751** | **-2.192** | **-2.732** | **-2.883** | **-2.872** | **-2.519** | **-1.921** | 0.026 |
|  | p-value | 0,802 |  | 0.312 | 0.451 | **0.000** | **0.000** | **0.000** | **0.000** | **0.000** | **0.000** | **0.000** | 0.932 |
| **FusG** | Coefficient | -0,386 | -0.311 |  | -0.082 | **-2.062** | **-2.502** | **-3.043** | **-3.194** | **-3.182** | **-2.830** | **-2.232** | -0.285 |
|  | p-value | 0,212 | 0.312 |  | 0.793 | **0.000** | **0.000** | **0.000** | **0.000** | **0.000** | **0.000** | **0.000** | 0.361 |
| **TC** | Coefficient | -0,304 | -0.229 | 0.082 |  | **-1.980** | **-2.421** | **-2.961** | **-3.112** | **-3.100** | **-2.748** | **-2.150** | -0.203 |
|  | p-value | 0,320 | 0.451 | 0.793 |  | **0.000** | **0.000** | **0.000** | **0.000** | **0.000** | **0.000** | **0.000** | 0.510 |
| **Amy** | Coefficient | **1,676** | **1.751** | **2.062** | **1.980** |  | -0.441 | **-0.981** | **-1.132** | **-1.120** | **-0.768** | -0.170 | **1.777** |
|  | p-value | **0,000** | **0.000** | **0.000** | **0.000** |  | 0.159 | **0.002** | **0.000** | **0.000** | **0.014** | 0.588 | **0.000** |
| **CA1** | Coefficient | **2,117** | **2.192** | **2.502** | **2.421** | 0.441 |  | -0.541 | **-0.691** | **-0.680** | -0.327 | 0.271 | **2.218** |
|  | p-value | **0,000** | **0.000** | **0.000** | **0.000** | 0.159 |  | 0.073 | **0.023** | **0.023** | 0.272 | 0.367 | **0.000** |
| **CA2** | Coefficient | **2,657** | **2.732** | **3.043** | **2.961** | **0.981** | 0.541 |  | -0.151 | -0.139 | 0.213 | **0.811** | **2.758** |
|  | p-value | **0,000** | **0.000** | **0.000** | **0.000** | **0.002** | 0.073 |  | 0.622 | 0.645 | 0.477 | **0.007** | **0.000** |
| **CA3** | Coefficient | **2,808** | **2.883** | **3.194** | **3.112** | **1.132** | **0.691** | 0.151 |  | 0.011 | 0.364 | **0.962** | **2.909** |
|  | p-value | **0,000** | **0.000** | **0.000** | **0.000** | **0.000** | **0.023** | 0.622 |  | 0.970 | 0.229 | **0.002** | **0.000** |
| **CA4** | Coefficient | **2,796** | **2.872** | **3.182** | **3.100** | **1.120** | **0.680** | 0.139 | -0.011 |  | 0.353 | **0.950** | **2.897** |
|  | p-value | **0,000** | **0.000** | **0.000** | **0.000** | **0.000** | **0.023** | 0.645 | 0.970 |  | 0.238 | **0.002** | **0.000** |
| **DG** | Coefficient | **2,444** | **2.519** | **2.830** | **2.748** | **0.768** | 0.327 | -0.213 | -0.364 | -0.353 |  | 0.598 | **2.545** |
|  | p-value | **0,000** | **0.000** | **0.000** | **0.000** | **0.014** | 0.272 | 0.477 | 0.229 | 0.238 |  | 0.045 | **0.000** |
| **Sub** | Coefficient | **1,846** | **1.921** | **2.232** | **2.150** | 0.170 | -0.271 | **-0.811** | **-0.962** | **-0.950** | -0.598 |  | **1.947** |
|  | p-value | **0,000** | **0.000** | **0.000** | **0.000** | 0.588 | 0.367 | **0.007** | **0.002** | **0.002** | 0.045 |  | **0.000** |
| **ParaSub** | Coefficient | -0,101 | -0.026 | 0.285 | 0.203 | **-1.777** | **-2.218** | **-2.758** | **-2.909** | **-2.897** | **-2.545** | **-1.947** |  |
|  | p-value | 0,741 | 0.932 | 0.361 | 0.510 | **0.000** | **0.000** | **0.000** | **0.000** | **0.000** | **0.000** | **0.000** |  |
| **p-tau load** | | **EntC** | **PHG** | **FusG** | **TC** | **Amy** | **CA1** | **CA2** | **CA3** | **CA4** | **DG** | **Sub** | **Para-Sub** |
| **EntC** | Coefficient |  | **9.256** | 3.528 | **-4.218** | 2.126 | **12.910** | **11.702** | -2.697 | **-6.352** | **-8.676** | 3.032 | **-7.942** |
|  | p-value |  | **0.000** | 0.046 | **0.016** | 0.264 | **0.000** | **0.000** | 0.128 | **0.000** | **0.000** | 0.081 | **0.000** |
| **PHG** | Coefficient | **-9,256** |  | **-5.728** | **-13.475** | **-7.130** | **3.654** | 2.446 | **-11.953** | **-15.608** | **-17.932** | **-6.225** | **-17.198** |
|  | p-value | **0,000** |  | **0.001** | **0.000** | **0.000** | **0.036** | 0.168 | **0.000** | **0.000** | **0.000** | **0.000** | **0.000** |
| **FusG** | Coefficient | -3,528 | **5.728** |  | **-7.747** | -1.402 | **9.382** | **8.174** | **-6.225** | **-9.880** | **-12.204** | -0.497 | **-11.470** |
|  | p-value | 0,046 | **0.001** |  | **0.000** | 0.464 | **0.000** | **0.000** | **0.000** | **0.000** | **0.000** | 0.777 | **0.000** |
| **TC** | Coefficient | **4,218** | **13.475** | **7.747** |  | **6.345** | **17.128** | **15.921** | 1.522 | -2.133 | **-4.458** | **7.250** | **-3.724** |
|  | p-value | **0,016** | **0.000** | **0.000** |  | **0.001** | **0.000** | **0.000** | 0.391 | 0.221 | **0.011** | **0.000** | **0.034** |
| **Amy** | Coefficient | -2,126 | **7.130** | 1.402 | **-6.345** |  | **10.784** | **9.576** | **-4.823** | **-8.478** | **-10.802** | 0.905 | **-10.068** |
|  | p-value | 0,264 | **0.000** | 0.464 | **0.001** |  | **0.000** | **0.000** | **0.012** | **0.000** | **0.000** | 0.632 | **0.000** |
| **CA1** | Coefficient | **-12,910** | **-3.654** | **-9.382** | **-17.128** | **-10.784** |  | -1.208 | **-15.607** | **-19.262** | **-21.586** | **-9.878** | **-20.852** |
|  | p-value | **0,000** | **0.036** | **0.000** | **0.000** | **0.000** |  | 0.493 | **0.000** | **0.000** | **0.000** | **0.000** | **0.000** |
| **CA2** | Coefficient | **-11,702** | -2.446 | **-8.174** | **-15.921** | **-9.576** | 1.208 |  | **-14.399** | **-18.054** | **-20.378** | **-8.671** | **-19.645** |
|  | p-value | **0,000** | 0.168 | **0.000** | **0.000** | **0.000** | 0.493 |  | **0.000** | **0.000** | **0.000** | **0.000** | **0.000** |
| **CA3** | Coefficient | 2,697 | **11.953** | **6.225** | -1.522 | **4.823** | **15.607** | **14.399** |  | **-3.655** | **-5.979** | **5.728** | **-5.245** |
|  | p-value | 0,128 | **0.000** | **0.000** | 0.391 | **0.012** | **0.000** | **0.000** |  | **0.038** | **0.001** | **0.001** | **0.003** |
| **CA4** | Coefficient | **6,352** | **15.608** | **9.880** | 2.133 | **8.478** | **19.262** | **18.054** | 3.655 |  | -2.324 | **9.383** | -1.591 |
|  | p-value | **0,000** | **0.000** | **0.000** | 0.221 | **0.000** | **0.000** | **0.000** | 0.038 |  | 0.178 | **0.000** | 0.362 |
| **DG** | Coefficient | **8,676** | **17.932** | **12.204** | **4.458** | **10.802** | **21.586** | **20.378** | **5.979** | 2.324 |  | **11.708** | 0.734 |
|  | p-value | **0,000** | **0.000** | **0.000** | **0.011** | **0.000** | **0.000** | **0.000** | **0.001** | 0.178 |  | **0.000** | 0.674 |
| **Sub** | Coefficient | -3,032 | **6.225** | 0.497 | **-7.250** | -0.905 | **9.878** | **8.671** | **-5.728** | **-9.383** | **-11.708** |  | **-10.974** |
|  | p-value | 0,081 | **0.000** | 0.777 | **0.000** | 0.632 | **0.000** | **0.000** | **0.001** | **0.000** | **0.000** |  | **0.000** |
| **ParaSub** | Coefficient | **7,942** | **17.198** | **11.470** | **3.724** | **10.068** | **20.852** | **19.645** | **5.245** | 1.591 | -0.734 | **10.974** |  |
|  | p-value | **0,000** | **0.000** | **0.000** | **0.034** | **0.000** | **0.000** | **0.000** | **0.003** | 0.362 | 0.674 | **0.000** |  |
| **Iba1 load** | | **EntC** | **PHG** | **FusG** | **TC** | **Amy** | **CA1** | **CA2** | **CA3** | **CA4** | **DG** | **Sub** | **Para-Sub** |
| **EntC** | Coefficient |  | **-0.488** | **-0.632** | **-0.526** | -0.253 | 0.138 | **1.334** | 0.376 | -0.100 | 0.209 | -0.064 | 0.084 |
|  | p-value |  | **0.017** | **0.003** | **0.011** | 0.230 | 0.499 | **0.000** | 0.072 | 0.625 | 0.310 | 0.756 | 0.688 |
| **PHG** | Coefficient | **0,488** |  | -0.144 | -0.038 | 0.236 | **0.627** | **1.822** | **0.864** | 0.388 | **0.697** | 0.424 | **0.572** |
|  | p-value | **0,017** |  | 0.497 | 0.855 | 0.261 | **0.002** | **0.000** | **0.000** | 0.058 | **0.001** | 0.040 | **0.006** |
| **FusG** | Coefficient | **0,632** | 0.144 |  | 0.107 | 0.380 | **0.771** | **1.966** | **1.008** | **0.532** | **0.841** | **0.568** | **0.716** |
|  | p-value | **0,003** | 0.497 |  | 0.617 | 0.081 | **0.000** | **0.000** | **0.000** | **0.012** | **0.000** | **0.008** | **0.001** |
| **TC** | Coefficient | **0,526** | 0.038 | -0.107 |  | 0.273 | **0.664** | **1.859** | **0.902** | 0.425 | **0.734** | **0.461** | **0.610** |
|  | p-value | **0,011** | 0.855 | 0.617 |  | 0.192 | **0.001** | **0.000** | **0.000** | 0.038 | **0.000** | **0.026** | **0.003** |
| **Amy** | Coefficient | 0,253 | -0.236 | -0.380 | -0.273 |  | 0.391 | **1.586** | **0.629** | 0.152 | 0.461 | 0.188 | 0.336 |
|  | p-value | 0,230 | 0.261 | 0.081 | 0.192 |  | 0.061 | **0.000** | **0.003** | 0.468 | 0.028 | 0.373 | 0.114 |
| **CA1** | Coefficient | -0,138 | **-0.627** | **-0.771** | **-0.664** | -0.391 |  | **1.195** | 0.238 | -0.239 | 0.070 | -0.203 | -0.055 |
|  | p-value | 0,499 | **0.002** | **0.000** | **0.001** | 0.061 |  | **0.000** | 0.251 | 0.242 | 0.730 | 0.324 | 0.792 |
| **CA2** | Coefficient | **-1,334** | **-1.822** | **-1.966** | **-1.859** | **-1.586** | **-1.195** |  | **-0.958** | **-1.434** | **-1.125** | **-1.398** | **-1.250** |
|  | p-value | **0,000** | **0.000** | **0.000** | **0.000** | **0.000** | **0.000** |  | **0.000** | **0.000** | **0.000** | **0.000** | **0.000** |
| **CA3** | Coefficient | -0,376 | **-0.864** | **-1.008** | **-0.902** | **-0.629** | -0.238 | **0.958** |  | **-0.476** | -0.167 | -0.440 | -0.292 |
|  | p-value | 0,072 | **0.000** | **0.000** | **0.000** | **0.003** | 0.251 | **0.000** |  | **0.022** | 0.421 | 0.036 | 0.166 |
| **CA4** | Coefficient | 0,100 | -0.388 | **-0.532** | -0.425 | -0.152 | 0.239 | **1.434** | 0.476 |  | 0.309 | 0.036 | 0.184 |
|  | p-value | 0,625 | 0.058 | **0.012** | 0.038 | 0.468 | 0.242 | **0.000** | 0.022 |  | 0.131 | 0.861 | 0.376 |
| **DG** | Coefficient | -0,209 | **-0.697** | **-0.841** | **-0.734** | -0.461 | -0.070 | **1.125** | 0.167 | -0.309 |  | -0.273 | -0.125 |
|  | p-value | 0,310 | **0.001** | **0.000** | **0.000** | 0.028 | 0.730 | **0.000** | 0.421 | 0.131 |  | 0.186 | 0.548 |
| **Sub** | Coefficient | 0,064 | -0.424 | **-0.568** | -0.461 | -0.188 | 0.203 | **1.398** | 0.440 | -0.036 | 0.273 |  | 0.148 |
|  | p-value | 0,756 | 0.040 | **0.008** | 0.026 | 0.373 | 0.324 | **0.000** | 0.036 | 0.861 | 0.186 |  | 0.479 |
| **ParaSub** | Coefficient | -0,084 | **-0.572** | **-0.716** | **-0.610** | -0.336 | 0.055 | **1.250** | 0.292 | -0.184 | 0.125 | -0.148 |  |
|  | p-value | 0,688 | **0.006** | **0.001** | **0.003** | 0.114 | 0.792 | **0.000** | 0.166 | 0.376 | 0.548 | 0.479 |  |
| **HLA-DR load** | | **EntC** | **PHG** | **FusG** | **TC** | **Amy** | **CA1** | **CA2** | **CA3** | **CA4** | **DG** | **Sub** | **Para-Sub** |
| **EntC** | Coefficient |  | 0.182 | -0.430 | -0.292 | **3.507** | **1.216** | **1.948** | **1.049** | **0.888** | 0.379 | **1.118** | -0.057 |
|  | p-value |  | 0.576 | 0.202 | 0.363 | **0.000** | **0.000** | **0.000** | **0.001** | **0.007** | 0.249 | **0.001** | 0.860 |
| **PHG** | Coefficient | -0,182 |  | -0.612 | -0.474 | **3.326** | **1.034** | **1.766** | **0.867** | 0.706 | 0.197 | **0.936** | -0.239 |
|  | p-value | 0,576 |  | 0.068 | 0.139 | **0.000** | **0.001** | **0.000** | **0.008** | 0.030 | 0.547 | **0.004** | 0.460 |
| **FusG** | Coefficient | 0,430 | 0.612 |  | 0.138 | **3.938** | **1.646** | **2.378** | **1.479** | **1.318** | **0.809** | **1.548** | 0.373 |
|  | p-value | 0,202 | 0.068 |  | 0.679 | **0.000** | **0.000** | **0.000** | **0.000** | **0.000** | **0.017** | **0.000** | 0.267 |
| **TC** | Coefficient | 0,292 | 0.474 | -0.138 |  | **3.800** | **1.508** | **2.240** | **1.342** | **1.180** | 0.671 | **1.410** | 0.235 |
|  | p-value | 0,363 | 0.139 | 0.679 |  | **0.000** | **0.000** | **0.000** | **0.000** | **0.000** | 0.038 | **0.000** | 0.463 |
| **Amy** | Coefficient | **-3,507** | **-3.326** | **-3.938** | **-3.800** |  | **-2.292** | **-1.560** | **-2.458** | **-2.619** | **-3.129** | **-2.390** | **-3.565** |
|  | p-value | **0,000** | **0.000** | **0.000** | **0.000** |  | **0.000** | **0.000** | **0.000** | **0.000** | **0.000** | **0.000** | **0.000** |
| **CA1** | Coefficient | **-1,216** | **-1.034** | **-1.646** | **-1.508** | **2.292** |  | **0.732** | -0.166 | -0.328 | **-0.837** | -0.098 | **-1.273** |
|  | p-value | **0,000** | **0.001** | **0.000** | **0.000** | **0.000** |  | **0.023** | 0.610 | 0.311 | **0.010** | 0.759 | **0.000** |
| **CA2** | Coefficient | **-1,948** | **-1.766** | **-2.378** | **-2.240** | **1.560** | **-0.732** |  | **-0.898** | **-1.060** | **-1.569** | **-0.830** | **-2.005** |
|  | p-value | **0,000** | **0.000** | **0.000** | **0.000** | **0.000** | **0.023** |  | **0.006** | **0.001** | **0.000** | **0.010** | **0.000** |
| **CA3** | Coefficient | **-1,049** | **-0.867** | **-1.479** | **-1.342** | **2.458** | 0.166 | **0.898** |  | -0.161 | -0.670 | 0.069 | **-1.106** |
|  | p-value | **0,001** | **0.008** | **0.000** | **0.000** | **0.000** | 0.610 | **0.006** |  | 0.626 | 0.044 | 0.833 | **0.001** |
| **CA4** | Coefficient | **-0,888** | **-0.706** | **-1.318** | **-1.180** | **2.619** | 0.328 | **1.060** | 0.161 |  | -0.509 | 0.230 | **-0.945** |
|  | p-value | **0,007** | **0.030** | **0.000** | **0.000** | **0.000** | 0.311 | **0.001** | 0.626 |  | 0.122 | 0.477 | **0.004** |
| **DG** | Coefficient | -0,379 | -0.197 | **-0.809** | -0.671 | **3.129** | **0.837** | **1.569** | 0.670 | 0.509 |  | **0.739** | -0.436 |
|  | p-value | 0,249 | 0.547 | **0.017** | 0.038 | **0.000** | **0.010** | **0.000** | 0.044 | 0.122 |  | **0.023** | 0.183 |
| **Sub** | Coefficient | **-1,118** | **-0.936** | **-1.548** | **-1.410** | **2.390** | 0.098 | **0.830** | -0.069 | -0.230 | **-0.739** |  | **-1.175** |
|  | p-value | **0,001** | **0.004** | **0.000** | **0.000** | **0.000** | 0.759 | **0.010** | 0.833 | 0.477 | **0.023** |  | **0.000** |
| **ParaSub** | Coefficient | 0,057 | 0.239 | -0.373 | -0.235 | **3.565** | **1.273** | **2.005** | **1.106** | **0.945** | 0.436 | **1.175** |  |
|  | p-value | 0,860 | 0.460 | 0.267 | 0.463 | **0.000** | **0.000** | **0.000** | **0.001** | **0.004** | 0.183 | **0.000** |  |
| **CD68 load** | | **EntC** | **PHG** | **FusG** | **TC** | **Amy** | **CA1** | **CA2** | **CA3** | **CA4** | **DG** | **Sub** | **Para-Sub** |
| **EntC** | Coefficient |  | 0.050 | -0.033 | **-0.152** | **0.331** | **0.345** | **0.589** | **0.319** | **0.243** | -0.016 | **0.358** | 0.114 |
|  | p-value |  | 0.360 | 0.561 | **0.005** | **0.000** | **0.000** | **0.000** | **0.000** | **0.000** | 0.769 | **0.000** | 0.039 |
| **PHG** | Coefficient | -0,050 |  | -0.083 | **-0.202** | **0.281** | **0.295** | **0.539** | **0.269** | **0.193** | -0.066 | **0.308** | 0.064 |
|  | p-value | 0,360 |  | 0.140 | **0.000** | **0.000** | **0.000** | **0.000** | **0.000** | **0.000** | 0.226 | **0.000** | 0.246 |
| **FusG** | Coefficient | 0,033 | 0.083 |  | **-0.119** | **0.363** | **0.378** | **0.622** | **0.352** | **0.276** | 0.017 | **0.390** | **0.146** |
|  | p-value | 0,561 | 0.140 |  | **0.033** | **0.000** | **0.000** | **0.000** | **0.000** | **0.000** | 0.766 | **0.000** | **0.009** |
| **TC** | Coefficient | **0,152** | **0.202** | **0.119** |  | **0.483** | **0.497** | **0.741** | **0.471** | **0.395** | **0.136** | **0.510** | **0.266** |
|  | p-value | **0,005** | **0.000** | **0.033** |  | **0.000** | **0.000** | **0.000** | **0.000** | **0.000** | **0.012** | **0.000** | **0.000** |
| **Amy** | Coefficient | **-0,331** | **-0.281** | **-0.363** | **-0.483** |  | 0.015 | **0.258** | -0.012 | -0.088 | **-0.347** | 0.027 | **-0.217** |
|  | p-value | **0,000** | **0.000** | **0.000** | **0.000** |  | 0.792 | **0.000** | 0.838 | 0.116 | **0.000** | 0.626 | **0.000** |
| **CA1** | Coefficient | **-0,345** | **-0.295** | **-0.378** | **-0.497** | -0.015 |  | **0.244** | -0.026 | -0.102 | **-0.361** | 0.012 | **-0.232** |
|  | p-value | **0,000** | **0.000** | **0.000** | **0.000** | 0.792 |  | **0.000** | 0.634 | 0.060 | **0.000** | 0.820 | **0.000** |
| **CA2** | Coefficient | **-0,589** | **-0.539** | **-0.622** | **-0.741** | **-0.258** | **-0.244** |  | **-0.270** | **-0.346** | **-0.605** | **-0.231** | **-0.475** |
|  | p-value | **0,000** | **0.000** | **0.000** | **0.000** | **0.000** | **0.000** |  | **0.000** | **0.000** | **0.000** | **0.000** | **0.000** |
| **CA3** | Coefficient | **-0,319** | **-0.269** | **-0.352** | **-0.471** | 0.012 | 0.026 | **0.270** |  | -0.076 | **-0.335** | 0.039 | **-0.205** |
|  | p-value | **0,000** | **0.000** | **0.000** | **0.000** | 0.838 | 0.634 | **0.000** |  | 0.167 | **0.000** | 0.483 | **0.000** |
| **CA4** | Coefficient | **-0,243** | **-0.193** | **-0.276** | **-0.395** | 0.088 | 0.102 | **0.346** | 0.076 |  | **-0.259** | **0.115** | **-0.129** |
|  | p-value | **0,000** | **0.000** | **0.000** | **0.000** | 0.116 | 0.060 | **0.000** | 0.167 |  | **0.000** | **0.034** | **0.018** |
| **DG** | Coefficient | 0,016 | 0.066 | -0.017 | **-0.136** | **0.347** | **0.361** | **0.605** | **0.335** | **0.259** |  | **0.374** | **0.130** |
|  | p-value | 0,769 | 0.226 | 0.766 | **0.012** | **0.000** | **0.000** | **0.000** | **0.000** | **0.000** |  | **0.000** | **0.018** |
| **Sub** | Coefficient | **-0,358** | **-0.308** | **-0.390** | **-0.510** | -0.027 | -0.012 | **0.231** | -0.039 | -0.115 | **-0.374** |  | -0.244 |
|  | p-value | **0,000** | **0.000** | **0.000** | **0.000** | 0.626 | 0.820 | **0.000** | 0.483 | 0.034 | **0.000** |  | 0.000 |
| **ParaSub** | Coefficient | **-0,114** | -0.064 | **-0.146** | **-0.266** | **0.217** | **0.232** | **0.475** | **0.205** | **0.129** | **-0.130** | **0.244** |  |
|  | p-value | **0,039** | 0.246 | **0.009** | **0.000** | **0.000** | **0.000** | **0.000** | **0.000** | **0.018** | **0.018** | **0.000** |  |
| **GFAP load** | | **EntC** | **PHG** | **FusG** | **TC** | **Amy** | **CA1** | **CA2** | **CA3** | **CA4** | **DG** | **Sub** | **Para-Sub** |
| **EntC** | Coefficient |  | **-3.356** | **-4.094** | **-6.007** | **-6.354** | **-6.531** | **-5.905** | **-5.422** | 0.735 | -0.296 | **-2.845** | -1.006 |
|  | p-value |  | **-0.003** | **0.000** | **0.000** | **0.000** | **0.000** | **0.000** | **0.000** | 0.520 | 0.796 | **0.013** | 0.382 |
| **PHG** | Coefficient | **3,356** |  | -0.737 | **-2.650** | **-2.997** | **-3.174** | **-2.549** | -2.066 | **4.092** | **3.061** | 0.511 | 2.350 |
|  | p-value | **0,003** |  | 0.533 | **0.021** | **0.011** | **0.006** | **0.029** | 0.077 | **0.000** | **0.008** | 0.657 | 0.043 |
| **FusG** | Coefficient | **4,094** | 0.737 |  | -1.913 | -2.260 | -2.437 | -1.812 | -1.329 | **4.829** | **3.798** | 1.249 | 3.087 |
|  | p-value | **0,000** | 0.533 |  | 0.104 | 0.060 | 0.038 | 0.129 | 0.267 | **0.000** | **0.001** | 0.290 | 0.009 |
| **TC** | Coefficient | **6,007** | **2.650** | 1.913 |  | -0.347 | -0.524 | 0.102 | 0.584 | **6.742** | **5.711** | **3.162** | 5.000 |
|  | p-value | **0,000** | **0.021** | 0.104 |  | 0.763 | 0.645 | 0.930 | 0.615 | **0.000** | **0.000** | **0.006** | 0.000 |
| **Amy** | Coefficient | **6,354** | **2.997** | 2.260 | 0.347 |  | -0.177 | 0.448 | 0.931 | **7.089** | **6.058** | **3.509** | 5.347 |
|  | p-value | **0,000** | **0.011** | 0.060 | 0.763 |  | 0.879 | 0.705 | 0.434 | **0.000** | **0.000** | **0.003** | 0.000 |
| **CA1** | Coefficient | **6,531** | **3.174** | 2.437 | 0.524 | 0.177 |  | 0.625 | 1.108 | **7.266** | **6.235** | **3.686** | 5.524 |
|  | p-value | **0,000** | **0.006** | 0.038 | 0.645 | 0.879 |  | 0.589 | 0.340 | **0.000** | **0.000** | **0.001** | 0.000 |
| **CA2** | Coefficient | **5,905** | 2.549 | 1.812 | -0.102 | -0.448 | -0.625 |  | 0.483 | **6.641** | **5.609** | **3.060** | 4.899 |
|  | p-value | **0,000** | 0.029 | 0.129 | 0.930 | 0.705 | 0.589 |  | 0.681 | **0.000** | **0.000** | **0.008** | 0.000 |
| **CA3** | Coefficient | **5,422** | 2.066 | 1.329 | -0.584 | -0.931 | -1.108 | -0.483 |  | **6.158** | **5.127** | 2.577 | 4.416 |
|  | p-value | **0,000** | 0.077 | 0.267 | 0.615 | 0.434 | 0.340 | 0.681 |  | **0.000** | **0.000** | 0.027 | 0.000 |
| **CA4** | Coefficient | -0,735 | **-4.092** | **-4.829** | **-6.742** | **-7.089** | **-7.266** | **-6.641** | **-6.158** |  | -1.031 | **-3.581** | -1.742 |
|  | p-value | 0,520 | **0.000** | **0.000** | **0.000** | **0.000** | **0.000** | **0.000** | **0.000** |  | 0.368 | **0.002** | 0.131 |
| **DG** | Coefficient | 0,296 | **-3.061** | **-3.798** | **-5.711** | **-6.058** | **-6.235** | **-5.609** | **-5.127** | 1.031 |  | **-2.549** | -0.711 |
|  | p-value | 0,796 | **0.008** | **0.001** | **0.000** | **0.000** | **0.000** | **0.000** | **0.000** | 0.368 |  | **0.026** | 0.538 |
| **Sub** | Coefficient | **2,845** | -0.511 | -1.249 | **-3.162** | **-3.509** | **-3.686** | **-3.060** | **-2.577** | **3.581** | **2.549** |  | 1.839 |
|  | p-value | **0,013** | 0.657 | 0.290 | **0.006** | **0.003** | **0.001** | **0.008** | **0.027** | **0.002** | **0.026** |  | 0.112 |
| **ParaSub** | Coefficient | 1,006 | -2.350 | **-3.087** | **-5.000** | **-5.347** | **-5.524** | **-4.899** | **-4.416** | 1.742 | 0.711 | -1.839 |  |
|  | p-value | 0,382 | 0.043 | **0.009** | **0.000** | **0.000** | **0.000** | **0.000** | **0.000** | 0.131 | 0.538 | 0.112 |  |

**Supplement 8.** Morphology of microglia and astroglia compared between limbic and cortical regions in pure and mixed DLB+AD. Representative images of microglial (**a-f, h-m, o-t**) and astrocytic (**v-A)** morphology in the PHG, CA1 and amygdala with a scatter plot of mean microglial and astroglial load [SD] of each marker in pure DLB and mixed DLB+AD cases (**g, n, u, B)**. **a, c, e, h, j, l, o, q, s** Many homeostatic and some small amoeboid microglial structures were observed in pure DLB cases. **b, d, f, i, k, m, p, r, t** Many large amoeboid and reactive microglial cells with short processes were observed in mixed DLB+AD cases. No regional differences were observed besides a higher load of microglial cells in limbic regions. **v, x, z** Astrocytes with a small cell soma and long, thin processes were observed in pure DLB cases. **w, y, A** In mixed DLB+AD cases, some astrocytes had a larger soma with long, thin processes. Between regions, no morphological heterogeneity between astrocytes was observed. Scale bar in **A** is identical for all images and represents 100 um. CA = cornu ammonis.


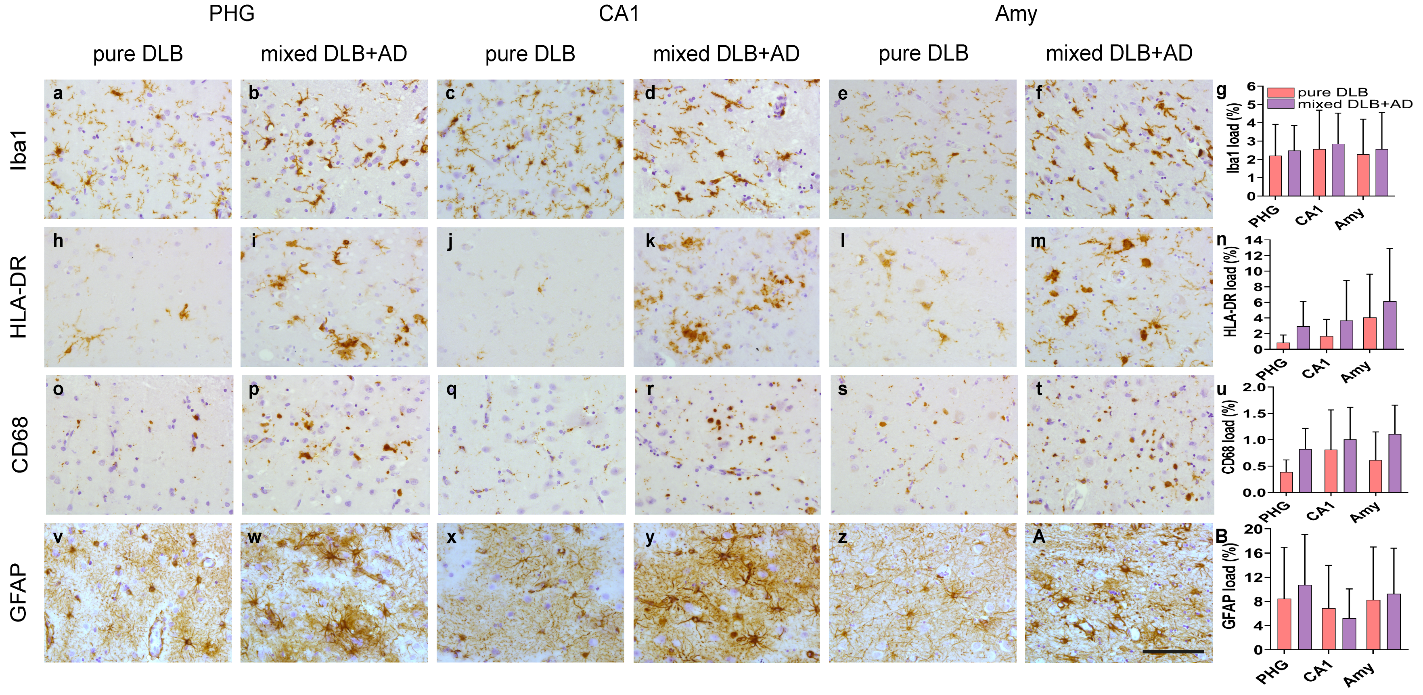


|  | x | | **IBA1** | **HLA-DR** | **CD68** | **GFAP** |  |
| --- | --- | --- | --- | --- | --- | --- | --- |
| **EntC** | **α-syn** | -0.13 | | 0.04 | 0.37 | 0.09 | -1 |
|  | **Aβ** | 0.04 | | 0.16 | 0.00 | -0.08 | 0 |
|  | **p-tau** | -0.06 | | -0.11 | 0.37 | -0.08 | 1 |
| **PHG** | **α-syn** | 0.07 | | 0.08 | 0.17 | -0.21 |  |
|  | **Aβ** | 0.07 | | 0.53 | 0.17 | 0.01 |  |
|  | **p-tau** | 0.04 | | -0.18 | **0.40**** | 0.05 |  |
| **FusG** | **α-syn** | 0.02 | | 0.18 | 0.28 | 0.19 |  |
|  | **Aβ** | -0.04 | | 0.11 | 0.21 | -0.17 |  |
|  | **p-tau** | 0.08 | | -0.15 | 0.45 | 0.07 |  |
| **TC** | **α-syn** | -0.14 | | 0.35 | 0.04 | 0.03 |  |
|  | **Aβ** | 0.18 | | 0.27 | 0.08 | 0.01 |  |
|  | **p-tau** | 0.32 | | 0.02 | **0.71***** | 0.23 |  |
| **Amy** | **α-syn** | 0.11 | | 0.03 | 0.06 | **0.68***** |  |
|  | **Aβ** | -0.06 | | 0.15 | 0.02 | 0.05 |  |
|  | **p-tau** | -0.05 | | 0.13 | 0.05 | 0.28 |  |
| **CA1** | **α-syn** | 0.05 | | **0.54*** | 0.08 | -0.04 |  |
|  | **Aβ** | 0.28 | | -0.12 | 0.35 | 0.29 |  |
|  | **p-tau** | 0.06 | | 0.30 | 0.29 | -0.11 |  |
| **CA2** | **α-syn** | 0.15 | | 0.27 | 0.30 | 0.38 |  |
|  | **Aβ** | -0.01 | | -0.30 | 0.02 | 0.31 |  |
|  | **p-tau** | 0.02 | | 0.07 | 0.33 | 0.06 |  |
| **CA3** | **α-syn** | 0.15 | | -0.12 | 0.19 | 0.25 |  |
|  | **Aβ** | 0.35 | | -0.14 | -0.46 | -0.17 |  |
|  | **p-tau** | 0.09 | | -0.14 | 0.36 | 0.17 |  |
| **CA4** | **α-syn** | 0.05 | | -0.14 | -0.06 | 0.04 |  |
|  | **Aβ** | -0.10 | | 0.44 | -0.01 | 0.37 |  |
|  | **p-tau** | 0.17 | | -0.45 | 0.21 | -0.23 |  |
| **DG** | **α-syn** | -0.16 | | -0.22 | -0.09 | 0.21 |  |
|  | **Aβ** | 0.21 | | 0.07 | 0.23 | -0.02 |  |
|  | **p-tau** | 0.16 | | -0.00 | 0.03 | -0.06 |  |
| **Sub** | **α-syn** | 0.09 | | 0.09 | 0.31 | 0.05 |  |
|  | **Aβ** | 0.10 | | -0.01 | 0.31 | -0.03 |  |
|  | **p-tau** | -0.04 | | 0.03 | 0.21 | -0.07 |  |
| **ParaSub** | **α-syn** | 0.02 | | 0.21 | -0.27 | -0.33 |  |
|  | **Aβ** | 0.05 | | 0.09 | 0.33 | -0.11 |  |
|  | **p-tau** | 0.04 | | -0.14 | 0.19 | -0.31 |  |

**Supplement 9.** Heatmap of standardized regression coefficients between microglial or astrocytic load and primary protein pathology load in mixed DLB+AD cases. Positive standardized regression coefficients are depicted in red, while negative standardized regression coefficients are denoted in blue in the heatmap. The regression coefficient is demonstrated in boxes and visualized in color scaling. Significance after FDR-correction is demonstrated in bold as * p < 0.05, ** p < 0.01, and *** p < 0.001.

**References**

1. Braak, H., Del Tredici, K., Rüb, U., de Vos, R. A., Jansen Steur, E. N., & Braak, E. (2003). **Staging of brain pathology related to sporadic Parkinson's disease**. *Neurobiol Aging,* 24(2), 197-211. doi:10.1016/s0197-4580(02)00065-9

2. Thal, D. R., Rüb, U., Orantes, M., & Braak, H. (2002). **Phases of A beta-deposition in the human brain and its relevance for the development of AD**. *Neurology,* 58(12), 1791-1800. doi:10.1212/wnl.58.12.1791

3. Braak, H., Alafuzoff, I., Arzberger, T., Kretzschmar, H., & Del Tredici, K. (2006). **Staging of Alzheimer disease-associated neurofibrillary pathology using paraffin sections and immunocytochemistry**. *Acta Neuropathol,* 112(4), 389-404. doi:10.1007/s00401-006-0127-z

4. Mirra, S. S., Heyman, A., McKeel, D., Sumi, S. M., Crain, B. J., Brownlee, L. M., . . . Berg, L. (1991). **The Consortium to Establish a Registry for Alzheimer's Disease (CERAD). Part II. Standardization of the neuropathologic assessment of Alzheimer's disease**. *Neurology,* 41(4), 479-486. doi:10.1212/wnl.41.4.479

5. Thal, D. R., Griffin, W. S., de Vos, R. A., & Ghebremedhin, E. (2008). **Cerebral amyloid angiopathy and its relationship to Alzheimer's disease**. *Acta Neuropathol,* 115(6), 599-609. doi:10.1007/s00401-008-0366-2

6. Finney, C. A., Jones, N. M., & Morris, M. J. (2021). **A scalable, fully automated approach for regional quantification of immunohistochemical staining of astrocytes in the rat brain**. *J Neurosci Methods,* 348, 108994. doi:10.1016/j.jneumeth.2020.108994

7. Paolicelli, R. C., Sierra, A., Stevens, B., Tremblay, M. E., Aguzzi, A., Ajami, B., . . . Wyss-Coray, T. (2022). **Microglia states and nomenclature: A field at its crossroads**. *Neuron,* 110(21), 3458-3483. doi:10.1016/j.neuron.2022.10.020

8. Reddaway, J., Richardson, P. E., Bevan, R. J., Stoneman, J., & Palombo, M. (2023). **Microglial morphometric analysis: so many options, so little consistency**. *Front Neuroinform,* 17, 1211188. doi:10.3389/fninf.2023.1211188

9. Zhou, B., Zuo, Y. X., & Jiang, R. T. (2019). **Astrocyte morphology: Diversity, plasticity, and role in neurological diseases**. *CNS Neurosci Ther,* 25(6), 665-673. doi:10.1111/cns.13123
